# The crosstalk between senescence, tumor, and immunity: molecular mechanism and therapeutic opportunities

**DOI:** 10.1002/mco2.70048

**Published:** 2025-01-14

**Authors:** Zehua Wang, Chen Chen, Jiaoyu Ai, Yaping Gao, Lei Wang, Shurui Xia, Yongxu Jia, Yanru Qin

**Affiliations:** ^1^ Department of Oncology The First Affiliated Hospital of Zhengzhou University Zhengzhou China; ^2^ Department of Gastroenterology, The First Affiliated Hospital of Nanchang University Nanchang China

**Keywords:** cancer treatment, cellular senescence, immunity, SASP, tumor microenvironment, tumor progression

## Abstract

Cellular senescence is characterized by a stable cell cycle arrest and a hypersecretory, proinflammatory phenotype in response to various stress stimuli. Traditionally, this state has been viewed as a tumor‐suppressing mechanism that prevents the proliferation of damaged cells while activating the immune response for their clearance. However, senescence is increasingly recognized as a contributing factor to tumor progression. This dual role necessitates a careful evaluation of the beneficial and detrimental aspects of senescence within the tumor microenvironment (TME). Specifically, senescent cells display a unique senescence‐associated secretory phenotype that releases a diverse array of soluble factors affecting the TME. Furthermore, the impact of senescence on tumor–immune interaction is complex and often underappreciated. Senescent immune cells create an immunosuppressive TME favoring tumor progression. In contrast, senescent tumor cells could promote a transition from immune evasion to clearance. Given these intricate dynamics, therapies targeting senescence hold promise for advancing antitumor strategies. This review aims to summarize the dual effects of senescence on tumor progression, explore its influence on tumor–immune interactions, and discuss potential therapeutic strategies, alongside challenges and future directions. Understanding how senescence regulates antitumor immunity, along with new therapeutic interventions, is essential for managing tumor cell senescence and remodeling the immune microenvironment.

## Graphical abstract

Cellular senescence plays both tumor‐suppressive and tumor‐progressive effects. It is critical to weigh the balance of the “bright” and “dark” sides of senescence. Immune cell senescence is an important strategy for tumors to occur immune escape. By contrary, senescent tumor cells can reshape the immune microenvironment to boost immune clearance.

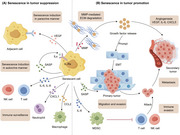

## 1 INTRODUCTION

Cellular senescence is a stress‐inducible state with four interdependent hallmarks: (1) an irreversible cell‐cycle arrest, (2) a senescence‐associated secretory phenotype (SASP), (3) alterations in morphology and physiology, and (4) a reprogrammed metabolism.^1^ In 1961, Hayflick and Moorhead first described cellular senescence in human diploid fibroblasts to explain the finite lifespan of normal cells, as these cells have achieved their maximum replicative capacity.^2^ This phenomenon was later shown to result from telomere shortening and defined as replicative senescence (RS).^3^ Therefore, cellular senescence plays a broad physiological role to maintain genome stability and avoid damage accumulation, especially during embryonic development, wound healing, tissue remodeling, and resolution of fibrosis.^4^, ^5^, ^6^, ^7^ Senescence can be triggered as a defense mechanism by various internal and external stressors. These include oncogenic, genotoxic, metabolic, lysosomal, and oxidative stress, which may result in different types of cellular senescence.^8^, ^9^, ^10^

Although much of our comprehension regarding the pathophysiological functions of senescent cells is grounded in experimental murine models and human somatic cells, emerging studies highlights the importance of cellular senescence in cancer biology and human disease. Specifically, several subsets of senescent cells might be associated with tumorigenesis, including oncogene‐induced senescence (OIS) and therapy‐induced senescence (TIS). Owing to their tumor‐promoting properties, cellular senescence is recently recognized as a hallmark of cancer.^11^

While both senescent cells and tumor cells involve the progressive accumulation of damaged cells, they exhibit diametrically opposed biological behavior. More specifically, tumor cells display the potential for hyperproliferation and elevated metabolic rates, whereas senescent cells exhibit change of functionality and a halt in cell division.^9^, ^12^ Additionally, emerging studies have proposed that cellular senescence may be a double‐edged sword in cancer, exerting pro‐ or antitumorigenic effects that depend on different contexts.^13^ Thereby, this dual effects of senescence significantly influences tumor progression or suppression.

The SASP, where senescent cells secrete a diverse set of proinflammatory cytokines, chemokines, growth factors, and proteinases, also plays an important role in the tumor microenvironment (TME).^14^ By releasing SASP factors, senescence can modulate pathways in neighboring cells and those at remote sites.^3^ Moreover, SASP factors are typically varied based on the type of senescent cells and the cellular milieu.^15^ Studies have shown that SASP factors can promote or inhibit tumor progression by altering the TME, which in turn affect the production of SASPs. This intricate interplay significantly influences how tumors develop. Though the mechanisms underlying this complex crosstalk are not completely clear, extensive preclinical studies have shown that modulating senescence or SASP holds promise for optimizing cancer therapy (more details in Section 5).

Immunity is characterized by the activation of various immune components, including T cells, B cells, natural killer (NK) cells, and antigen‐presenting cells (APCs), which work collectively to identify and destroy malignant cells. The antitumor immune response is initiated when tumor‐associated antigens, which are unique proteins expressed by cancer cells, are presented to the immune system. This recognition triggers a cascade of immune reactions aimed at eradicating the tumor.^16^, ^17^ The roles of immunity in the context of cellular senescence are ambiguous and even controversial. Senescence is a natural physiological process whereby organ function gradually deteriorates. When senescence occurs in the immune system, it is termed immunosenescence, characterized by immune dysfunction and lymphoid organ remodeling.^18^ It is a multifactorial phenomenon leading to high susceptibility to malignancy.^18^, ^19^, ^20^ Conversely, recent publications highlight that senescence might induce phenotypic changes in tumor cells, which subsequently stimulates the immune system and boosts the antitumor immunity.^21^ Given the complexity of immunity and tumor evolution, it is necessary to exceptionally convolute how senescent cells modulate antitumor immune response. Therapies that target tumor cell senescence or reverse immune cell senescence hold the potential to extend lifespan and are currently undergoing clinical trials.

In this review, we first introduce the induction process of cellular senescence, discuss the context‐dependent roles of senescence with both tumor‐suppressive and tumor‐promoting features, explore the complicated effects of senescence on tumor–immune crosstalk, provide an overview of therapies that inhibit the SASP (senomorphics) or selectively attack senescent tumor cells (senolytics), highlight promising strategies to prevent T‐cell senescence for boosting cancer immunotherapy, and conclude potential challenges and future directions in this field.

## 2 INDUCTION OF CELLULAR SENESCENCE

Cellular senescence could occur in response to various stressors, including DNA damage, oxidative stress, and telomere shortening. There are several types of cellular senescence, primarily classified based on their triggering stimuli and associated biological contexts. The most recognized forms involve RS and stress‐induced premature senescence. Senescence‐associated cell cycle arrest functions as a tumor suppressor, with its induction arising from the engagement of various signaling and downstream cell cycle inhibitor pathways.

### 2.1 Replicative senescence

RS is a fundamental biological process characterized by irreversible cessation of cell division, which occurs after a finite number of cell divisions in somatic cells.^2^, ^22^, ^23^, ^24^ Notably, a cell cycle arrest is required only for dividing cells, while nondividing and postmitotic cells can be senescent as well.^25^ RS is primarily driven by telomere shortening, a consequence of the end‐replication problem during DNA replication. Telomeres, the protective caps at the ends of chromosomes, shorten with each cell division due to the inability of DNA polymerase to fully replicate the ends of linear DNA. When telomeres reach a critical length, they are identified as DNA DSB, triggering a DNA damage response (DDR). The initial checkpoint kinases, ATM (ataxia‐telangiectasia mutated) and ATR (ataxia‐telangiectasia and Rad 3‐related) are subsequently activated to phosphorylate several proteins, with CHK2 being one of them. CHK2 transmits DDR signals by phosphorylating the tumor suppressor protein p53, which then activated the downstream protein p21. P21 prevents the phosphorylation of retinoblastoma protein (RB), leading to the cell cycle arrest at the G1 phase and enter cellular senescence.^26^, ^27^ In addition to the p53/p21 pathway, p16INK4a/RB could also induce RS.^28^

RS is also influenced by various molecular pathways and environmental factors. For instance, oxidative stress, often a byproduct of cellular metabolism, can induce DNA damage and contribute to the senescence phenotype. Reactive oxygen species (ROS) leads to genomic instability and further activates the DDR, exacerbating the senescence process.^29^ Furthermore, metabolic alterations, such as changes in serine metabolism, have been shown to regulate RS through epigenetic modifications like histone methylation, further complicating the molecular landscape of this process.^30^

Recent studies have also highlighted the role of RNA modifications, particularly m6A methylation, in the regulation of RS. Dynamic alterations in m6A methylation patterns have been observed during senescence, suggesting a regulatory mechanism that could influence gene expression and cellular aging.^31^ Additionally, the interaction of various signaling pathways, including the TGF‐β and mTOR pathways, has been implicated in the modulation of senescence, indicating that RS is not merely a result of telomere shortening but a complex interplay of genetic, epigenetic, and environmental factors.^32^, ^33^

### 2.2 Stress‐induced premature senescence

Numerous stressors could induce early senescence that can be classified into different categories based on the trigger. The following sections describe various forms of cancer‐related senescence.

#### 2.2.1 Oncogene‐induced senescence

OIS is primarily caused by the activation of oncogenes, leading to a state of permanent growth arrest, which was first demonstrated in vivo in 1997.^34^, ^35^, ^36^ Subsequently, the concept of OIS was expanded to multiple tumorigenesis models, including lung adenomas, lymphoma, prostate cancer, and melanocytic naevi.^34^, ^37^, ^38^, ^39^ OIS acts as a barrier to tumorigenesis by preventing the proliferation of cells that harbor oncogenic mutations. Notably, this process can be triggered by various oncogenes, such as RAS, BRAF, and MYC, each contributing to the complex landscape of cancer biology. For example, melanocytic nevi caused by oncogenic BRAF mutations keep senescent for decades, thus impeding their progression into melanoma.^39^ PTEN inactivation induces growth arrest through the p53‐dependent cellular senescence pathway in primary prostate epithelium.^37^

The mechanisms underlying OIS have been clarified. The activation of an oncogene triggers the production of ROS, introducing DSBs and DDR, which subsequently initiates cellular senescence.^40^ The activation of oncogenes results in DNA damage, which upregulates the expression of functional tumor suppressor proteins like p53 and p21. This upregulation induces a stable cell cycle arrest by inhibiting the downstream cyclin‐dependent kinase (CDK)–cyclin complexes. Thereby, hyperphosphorylation of RB protein blocks S‐phase entry and induces cellular senescence.^35^, ^41^, ^42^ Of note, only oncogene expression does not drive DDR in the absence of DNA replication; instead, OIS originates from DDR activation triggered by oncogene‐induced DNA hyper‐replication.^43^

#### 2.2.2 Therapy‐induced senescence

TIS is considered a reaction in tumor cells to an array of anticancer therapies, including chemotherapy and radiotherapy, which introduce DSBs and activate DDR.^44^

The mechanisms of TIS are complex and involve various signaling pathways and cellular responses. One of the key pathways implicated in TIS is the activation of the p53 and p16INK4a tumor suppressor pathways, which play critical roles in mediating cell cycle arrest and cellular senescence. Following DNA damage induced by chemotherapy or radiation, p53 is activated, leading to the transcription of genes that inhibit the cell cycle, thereby promoting senescence.^45^ For instance, cisplatin triggers the p53 pathway to induce cellular senescence.^46^ Additionally, an elevated level of ROS during therapy can further activate these pathways, exacerbating the senescence response.

Several studies have provided insights into the role of TIS in the context of specific cancer therapies. For instance, in the treatment of T‐cell acute lymphoblastic leukemia with chemotherapy, the induction of senescence was found to suppress disease progression, suggesting that TIS might be beneficial in this context.^47^ Research has demonstrated that targeting senescent cells can enhance the efficacy of cancer therapies. For example, the use of senolytic drugs, which selectively eliminate senescent cells, has shown promise in reducing the side effects associated with chemotherapy while improving overall treatment outcomes.^48^, ^49^ These findings underscore the importance of understanding TIS not only as a byproduct of cancer treatment but also as a potential target for therapeutic intervention.

## 3 EFFECTS OF SENESCENCE ON TUMOR CELLS

**FIGURE 1:**
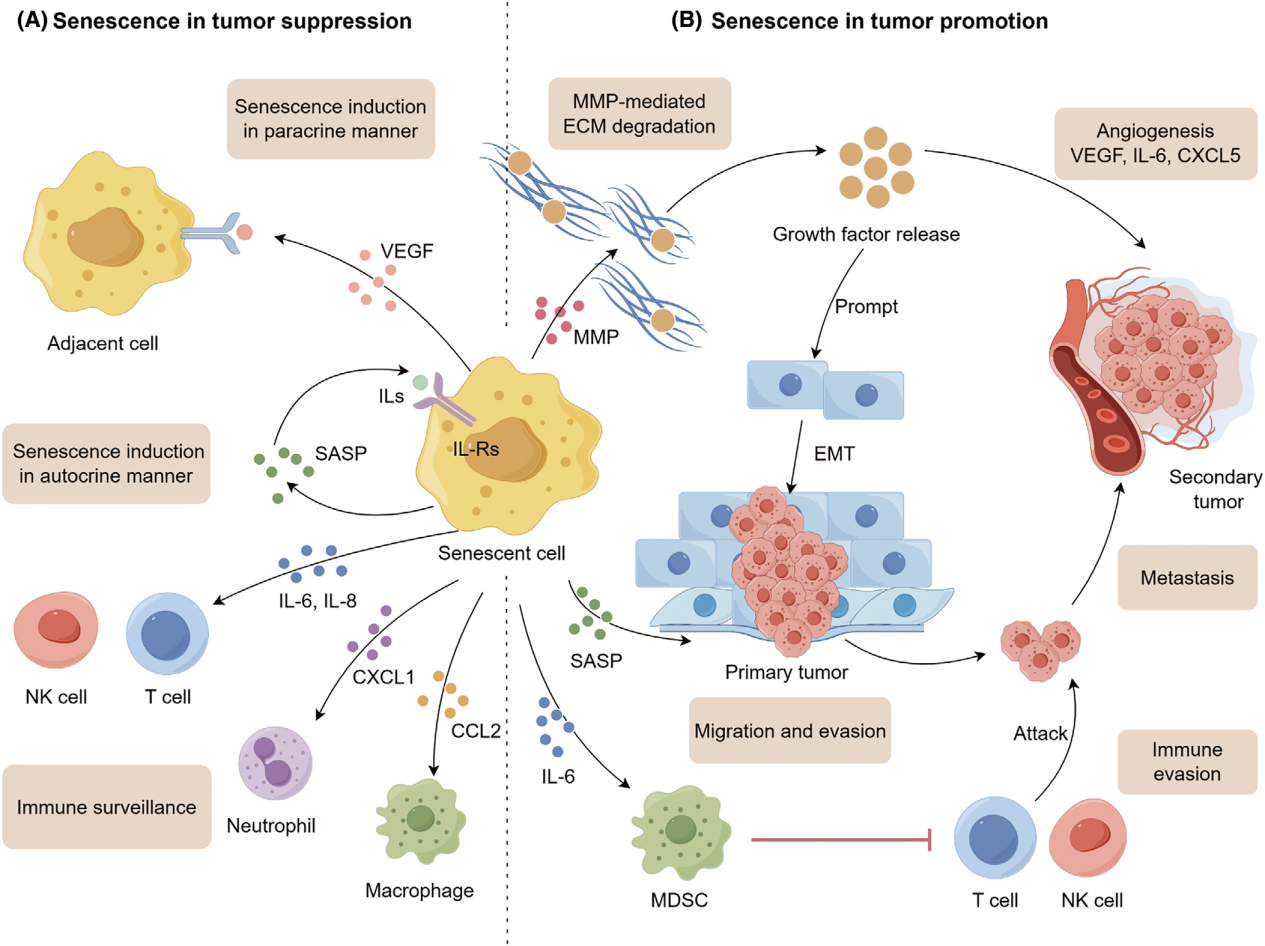
Effects of cellular senescence in tumor cell suppression and promotion. (A) SASP factors suppress tumor by reinforcing cell cycle arrest or enhancing immune surveillance. IL‐6 and IL‐8 can strengthen cellular senescence in an autocrine manner, while interleukins can spread senescence to adjacent cancer cells in a paracrine manner. Some cytokines, such as IL‐6, IL‐8, CXCL1, and CCL2, could recruit natural killer (NK) cell, T cell, neutrophils, and macrophages contributing to immune surveillance. (B) In the TME, various SASP factors are linked to cancer proliferation, migration, invasion, and metastasis, consequently enhancing the malignant capacities of cancer cell population. Also, several SASP factors could modulate the TME by prompting angiogenesis and preventing the antitumor functions of immune effectors. ECM, extracellular matrix; EMT, epithelial‐to‐mesenchymal transition; MDSC, myeloid‐derived suppressor cells; MMP, matrix metalloproteinase; SASP, senescence‐associated secretory phenotype; VEGF, vascular endothelial growth factor.

Cellular senescence is a double‐edged sword in cancer, exerting both protumorigenic and antitumorigenic effects (Figure 1). It is noted that senescent cells triggered by different stress stimuli may secrete diverse SASP components.^50^ On the one hand, SASP factors can reinforce cell cycle arrest in an autocrine scenario and mediate paracrine senescence to adjacent cells.^51^, ^52^, ^53^ Meanwhile, SASP factors are essential contributors to favor immune clearance via recruiting immune effectors.^54^, ^55^ On the other hand, the SASPs could remodel the TME beneficial for tumor cells to proliferate, invade, and metastasize while increasing immunosuppressive cell infiltration contributing to immune evasion.^15^, ^56^ Whether the effects of senescent cells are protumorigenic or antitumorigenic partly depends on the complicated interplay between various SASP factors and the tumor immune microenvironment (TIME). Table 1 summarizes the roles of different SASP factors in the context of specific cancer types.

**TABLE 1:** Different SASP factors in the tumor microenvironment.

	SASP factors	Cancer type	Senescent cell	Major roles of the SASP	References
Antitumorigenic SASP	IL‐1α	Liver cancer	Hepatocyte	Immune clearance of senescent tumor cell	^55^
	IL‐6	Osteosarcoma	Osteoblast	NKT cell recruitment	^57^
	CCL5	Melanoma	Melanocyte	Lymphocyte recruitment	^58^
	CCL2	Liver cancer	Hepatocyte	Macrophage activation	^54^
	VEGF	Pancreatic cancer	Pancreatic ductal cell	Enhanced vascularization; improved drug delivery efficacy	^59^
Protumorigenic SASP	CCL2	Liver cancer	Hepatocyte	MDSC differentiation	^54^
	PGE2	Liver cancer	Hepatic stellate cell	Impaired antitumor function of CD8+ T cell	^59^
	MMPs	–	Fibroblast	Tumor invasion promotion	^60^
	CXCL1, CXCL2	Prostate cancer	Prostate epithelial cell	Myeloid cell recruitment	^61^
	CXCL12	Thyroid cancer	Thyroid follicular cell	Anoikis resistance	^62^

Abbreviations: MDSC, myeloid‐derived suppressor cells; NK T cell, natural killer cell; SASP, senescence‐associated secretory phenotype.

### 3.1 Cellular senescence in tumor suppression

#### 3.1.1 Senescence‐mediated growth arrest

Entry of cells into senescence is a natural barrier to tumorigenesis, essential for eliminating cells from proliferation, particularly those with a heightened risk of malignant transformation.^23^ OIS operates as a cell‐intrinsic mechanism that induces growth arrest of premalignant cells following activated oncogenes (such as *N‐RAS^G12V^* and *BRAF^V600E^*), which could be further reinforced by CDK inhibitors (commonly known as p21 and p16^INK4a^).^34^, ^63^, ^64^ Senescence also mediates tumor suppression in a cell‐extrinsic fashion. SASP factors limit the propagation of precancerous cells or fully malignant cells in their vicinity through autocrine or paracrine manners.^51^, ^65^, ^66^ IL‐1α triggers an autocrine inflammatory response via activating NF‐κB signaling, driving the production of IL‐6 and IL‐8 that could reinforce growth arrest through increased ROS production and sustained DDR.^67^, ^68^ IL‐1α also stimulates paracrine senescence in adjacent cancer cells to prompt tumor suppression.^51^, ^65^, ^66^ Moreover, some SASP factors could induce apoptosis or necrosis in neighboring cells. TNF‐α triggers ROS‐dependent apoptosis in cancer cells.^69^ Conversely, IL‐6 drives apoptosis in tumor‐infiltrating T cells.^70^

#### 3.1.2 Senescence‐mediated immune surveillance

Provided evidence suggests that SASP is an important contributor to cancer immunosurveillance. Explicitly, IL‐6, IL‐8, and CCL2 facilitate the recruitment of M_1_‐type macrophages, T helper 1 cells, and NK cells to the TME, driving the immune clearance of senescent cancer cells.^54^, ^55^ Both OIS and TIS could trigger SASP‐mediated immunosurveillance against tumors.

Oncogene‐induced senescent cells have the capacity to promote cancer immunosurveillance. For instance, *N‐RAS^G12V^*‐driven senescent premalignant hepatocytes secrete SASP factors and are subject to immune elimination that depends on CD4^+^ T cell‐mediated adaptive immunity.^55^ When *NRAS^G12V^*‐induced senescent human fibroblasts are injected into mice, a global remodeling of their super‐enhancer landscape occurs. Super‐enhancers are particularly powerful enhancer regions in the genome that significantly boost gene expression. This reconfiguration, particularly through the recruitment of chromatin reader bromodomain‐containing protein 4 (BRD4) to SASP‐gene‐adjacent super‐enhancer loci within the genome, plays a pivotal role in immunosurveillance. Given that BRD4 is essential for the SASP factors and downstream paracrine signaling, BRD4 inhibition would destroy the immune clearance of premalignant OIS cells.^71^ Importantly, except for participating in the induction of cellular senescence, the tumor suppressor p53–p21 axis also modifies the interplay between senescence and immunity.^72^, ^73^ As a tumor suppressor, p53 is frequently mutated in various tumors, manifesting the “proliferating state” tumor with the worst prognosis.^74^, ^75^ P53‐restoration in mouse models contributes to tumor suppression with senescent phenotype, revealing that p53‐mediated senescence paves the way for immune clearance triggered by the SASP factors, such as CSF1, CCL2, CXCL1, and IL‐15.^72^, ^73^ By coordinating with NF‐κB signaling, p53 regulates SASP secretion that triggers macrophage activation contributing to a tumor‐suppressive microenvironment.^76^, ^77^ p21‐induced senescence also activates immunosurveillance with a particular secretory phenotype involving CXCL14 and IGFBP3 that prompt the recruitment of macrophages and lymphocytes into the TME.^78^ Moreover, oncogenic *RAS* was found to drive immune surveillance against tumorigenesis via p21‐dependent senescence.^78^

TIS also contributes to tumor suppression via immune cell‐mediated clearance. In the *KRAS*‐mutant lung cancer model, combinatory inhibitors of MEK and CDK4/6 suppress cancer cell proliferation while inducing *RB*‐mediated cellular senescence and activating immunomodulatory SASP. SASP components, TNF‐α and ICAM1, are needed for NK cell surveillance leading to tumor regression.^79^ Similarly, SASP factors lead to vascular remodeling that facilitates cytotoxic CD8^+^ T cell accumulation following dual inhibitors of MEK and CDK4/6 in *KRAS*‐mutant pancreatic ductal adenocarcinoma.^68^ Furthermore, CDK4/6 inhibitor‐mediated senescence induces potent immunosurveillance via regulatory T (Treg) cell suppression and CD8^+^ T cell enhancement.^67^, ^80^

### 3.2 Cellular senescence in tumor promotion

Though the SASP initially functions in tumor‐suppressive roles by reinforcing growth arrest and facilitating immunosurveillance, this phenotype exhibits paradoxical tumor‐promoting properties during ageing.^3^, ^81^, ^82^

#### 3.2.1 SASP‐driven carcinogenesis

Several studies have demonstrated protumorigenic effects of senescent cells mediated by individual SASP factors. Matrix metalloproteinases (MMPs) can mediate the degradation of extracellular matrix (ECM) components associated with growth factor release while prompting tumor‐driven angiogenesis, operating as a switch favoring tumor growth and invasion.^60^, ^83^, ^84^ Accordingly, GDF‐15, FGF1, and IGFBP3 also facilitate tumor dissemination to secondary sites.^85^ Moreover, hepatocyte growth factor (HGF) is reported to collaborate with MMPs to further promote tumor progression.^60^ SASP factors IL‐6 and IL‐8 are important drivers of cancer proliferation via creating a chronic inflammatory microenvironment, facilitating ECM cleavage by MMPs, and driving epithelial‐to‐mesenchymal transition (EMT), thereby collectively contributing to tumor invasiveness.^56^, ^86^, ^87^ Furthermore, VEGF, IL‐6, and CXCL5 stimulate angiogenesis to support tumor metastasis. Overall, different SASP factors mediate tumor growth, proliferation, invasion, and metastasis via multiple mechanisms.

#### 3.2.2 SASP‐mediated immune evasion

As a negative effect, SASP factors are critical mediators of TIME suppression leading to immune evasion. For example, IL‐6 is proven to recruit myeloid‐derived suppressor cells (MDSCs) to the tumor site, which are reported to block immune surveillance by suppressing CD8^+^ T cells and NK cells.^54^, ^88^ Meanwhile, MDSCs are found to inhibit IL‐1α signaling and consequently hinder the induction of senescence in tumor cells.^61^ IL‐6 and IL‐8 lead to the upregulation of HLA‐E that interacts with Natural Killer Group 2 Member A (NKG2A), which blunts the effector activity of NK cells and CD8^+^ T cells.^89^

#### 3.2.3 Senescence‐associated stemness

Pioneering studies pointed out that TIS can induce stemness properties in malignant cells, driving them to escape proliferation arrest and re‐enter the cell cycle.^90^, ^91^ For instance, chemotherapy‐induced senescence in transgenic mice with B‐cell lymphomas where WNT signaling is activated ultimately showed distinct stem‐cell markers.^90^ Oncogene‐induced senescent cells also acquire stemness characteristics driving tumor aggressiveness.^92^ In an OIS mouse model of breast cancer, RANK‐induced senescence is required for RANK‐triggered stemness; despite initially displaying a delayed tumor onset, tumor progression and aggressiveness are observed in the long term.^93^ Moreover, overexpression of CIP1 enables p53‐null tumor cells to gain enhanced stem cell properties following a transient senescent state.^94^ Growing evidence indicates that senescence‐associated stemness is triggered by WNT signaling activation depending on SASP factors rather than WNT ligands.^90^ Therefore, senescent tumor cells that regain proliferative capacity exhibit WNT‐dependent growth, a process that is particularly common in recurrent tumors.^90^

## 4 EFFECTS OF SENESCENCE ON TUMOR–IMMUNE CROSSTALK

The interaction between cellular senescence and antitumor immunity is complex. Explicitly, immune system senescence may reshape the TME by reducing the infiltration of immune effector cells and diminishing immune functions, facilitating tumor cells’ ability to evade immune surveillance. On the contrary, tumor cell senescence might trigger a switch from immune evasion to immune elimination.

### 4.1 Senescence‐related immune cells inducing an incompetent TIME

Immunosenescence proposed by Roy Walford in 1964,^18^ reflects the senescence of various immune cell subsets essential for antitumor activity. Since then, scientists have devoted a deeper insight into the mechanisms and effects of immunosenescence. In 2000, inflammaging was first introduced by Claudio Franceschi, a state of low‐grade, chronic damage resulting from high levels of proinflammatory markers within the body.^95^, ^96^ Inflammaging could result from immunosenescence‐mediated immune dysfunction but also engenders a feedforward process driving immunosenescence.^97^, ^98^ Emerging evidence supports that changes in immune cell subpopulations may trigger a shift toward an incompetent TIME.

#### 4.1.1 Abnormal molecular mechanisms underlie immunosenescence

**FIGURE 2:**
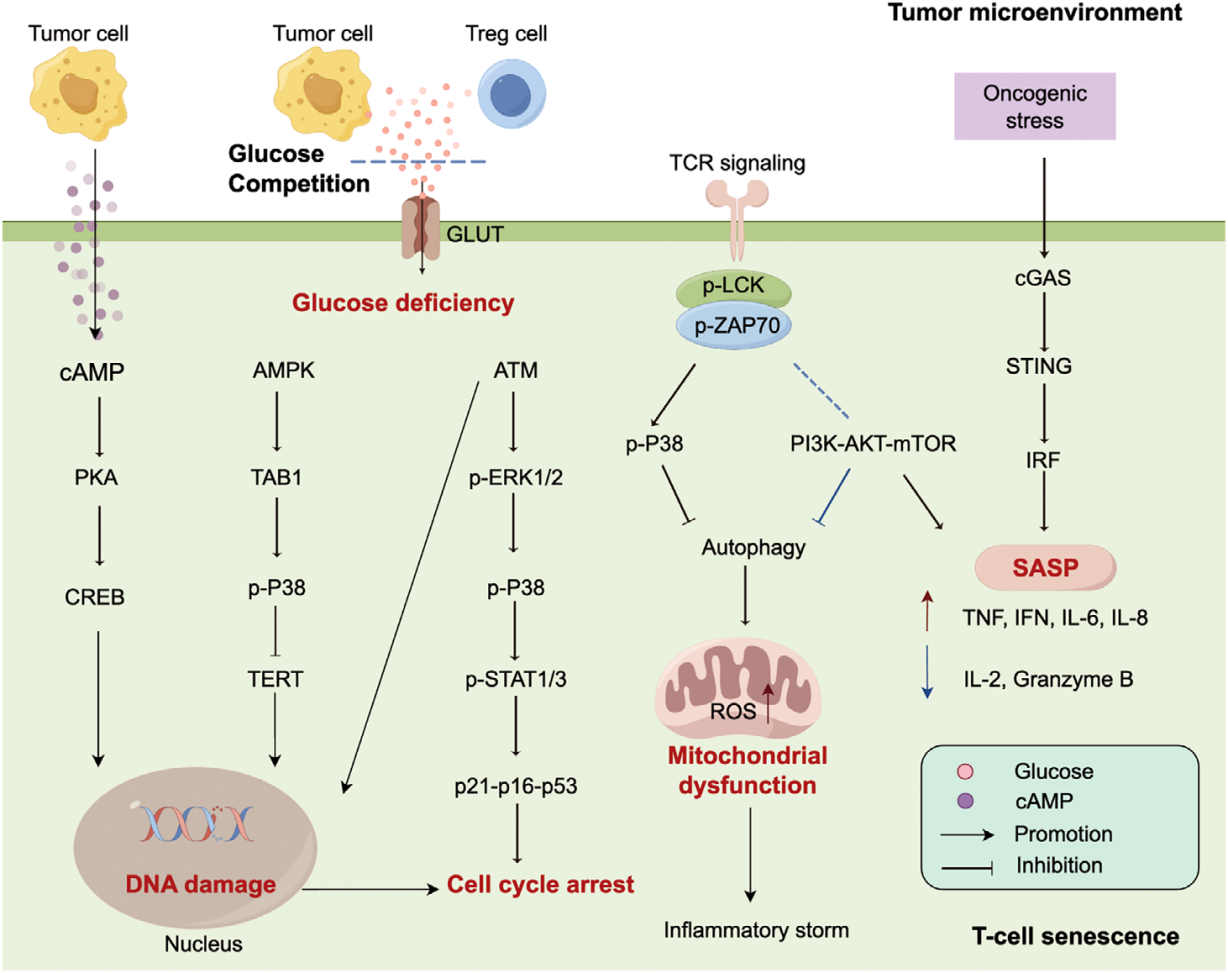
T cell senescence‐related signaling pathways. Hypoxia‐driven cAMP can be transferred to T cells, inducing DNA damage via PKA–CREB pathway activation. Glucose competition could trigger ATM‐related DNA damage, induce ERK1/2 and P38 pathways, and interacts with STAT1/3, leading to T‐cell senescence. The downregulation of TCR signaling can trigger the activation of P38 pathway while inhibiting the PI3K‐AKT–mTOR signaling pathway. This leads to the inactivation of autophagy and the induction of mitochondrial dysfunction in senescent T cells. The cGAS–STING pathway also modulates the secretion of SASP factors. Treg cell, regulatory T cell; TCR, T cell receptor; SASP, senescence‐associated secretory phenotype.

Recent studies have underscored the significance of immunosenescence in tumor development.^99^ Many factors within the TME possess the capability to trigger immunosenescence. As the most powerful immune cells in eradicating tumor cells, T‐cell senescence has attracted the most interest. Given the potential of T cells to be continuously activated by antigens and affected by inflammatory cytokines, it is plausible that the TME may be the source of senescent T cells.^100^ Signaling pathways involved in T‐cell senescence are displayed in Figure 2.

Tumor‐derived cyclic adenosine monophosphate (cAMP), driven by hypoxia, has the potential to inhibit tumor‐specific effector T cells by inducing DNA damage and senescence.^101^, ^102^ Glucose competition between T cells and Treg cells could initiate a series of events, including ATM‐related DNA damage, activation of ERK1/2 and P38 pathways, and interaction with STAT1/3 accompanied by upregulation of p21–p16–p53, eventually leading to T cells withdraw from the cell cycle.^103^, ^104^ Furthermore, the activation of AMPK pathways due to glucose deficiency could downregulate the telomerase reverse transcriptase gene binding to TAB1, followed by autophosphorylation of P38. The activation of P38 leads to the downregulation of TERT, thereby inducing DNA damage.^105^ The downregulation of T cell receptor (TCR) signaling could activate the P38 pathway and hinder the PI3K/AKT/mTOR signaling pathway, which in turn inactivates autophagy and accelerates ROS production in senescent T cells. Of note, high levels of ROS contribute to inflammaging and immunosenescence.^106^

#### 4.1.2 Cellular level mechanisms in immunosenescence

**FIGURE 3:**
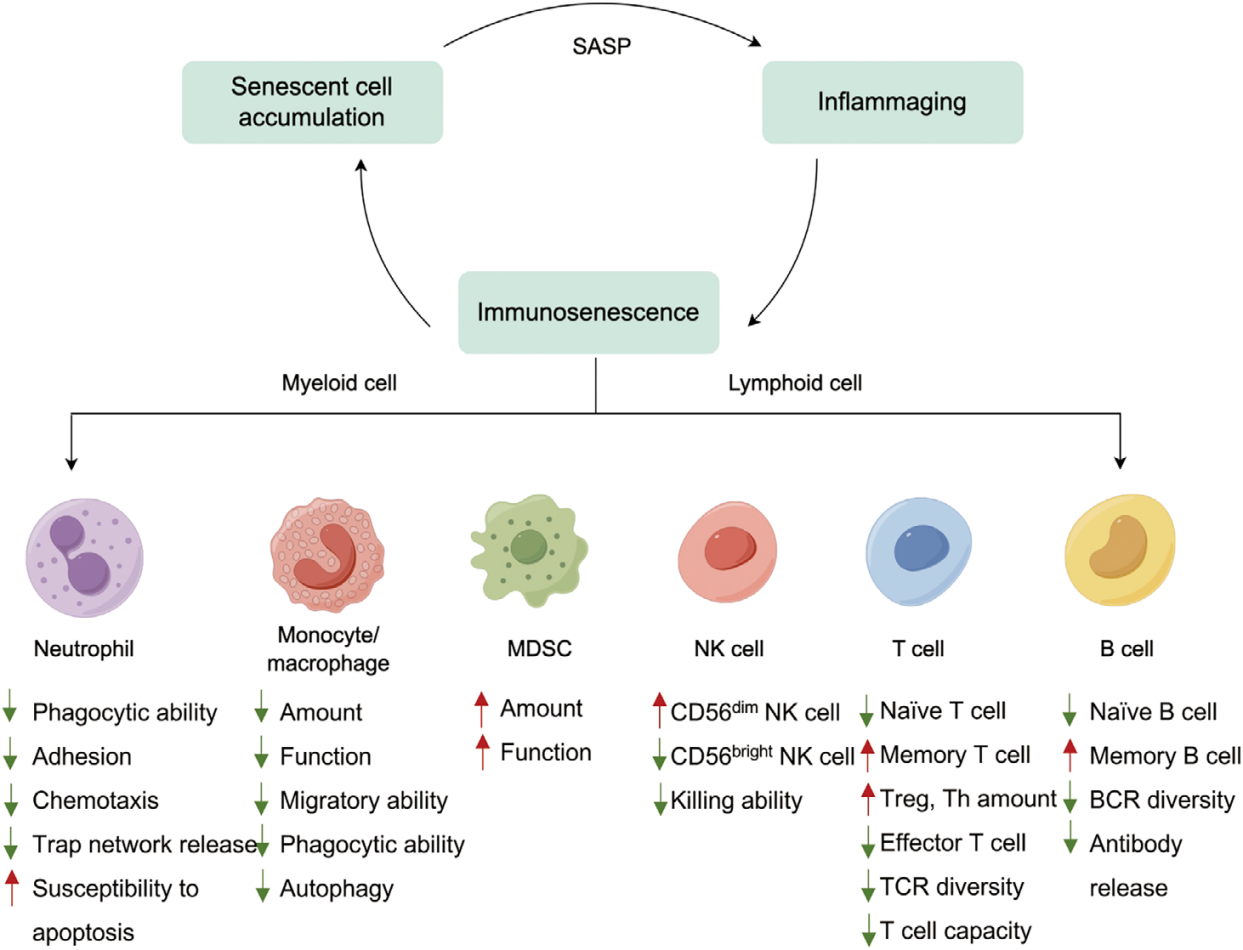
Major changes in different immune cell subsets during immunosenescence. The senescence of the immune system indicates aging of various immune cell types. Prominent characteristics of immune cell senescence include a decline in immune function and an increase in the secretion of inflammatory factors. The number of naïve T and B cell is decreased, while the quantity of memory T and B cell is increased. The antigen presentation and phagocytic functions of dendritic cells are impaired. The functionality of NK cells is impaired, while the number and activity of MDSCs and macrophages are increased. MDSCs, myeloid‐derived suppressor cells; NK cell, natural killer cell.

Immune system senescence, also referred to immunosenescence, reflects the senescence process observed in diverse subpopulations of immune cells (Figure 3). Innate immune senescence is characterized by a reduced ability to process and present antigens, leading to a diminished responsiveness to stimuli. Meanwhile, adaptive immune senescence encompasses a decline in the diversity of the TCR repertoire and an impaired formation of immunological memory. Therefore, immune cell senescence dramatically impacts the efficacy of antitumor responses.

##### T cells

Senescent T cells, with defects in effector function, accumulate in the context of ageing, chronic inflammation, and autoimmune disorders where antigen stimulation persistently exists.^107^, ^108^ Numerous cytokines and signals that coexist in the TME could induce T cell senescence, paving the way for tumorigenesis and cancer progression.^100^

Various T‐cell subpopulations display heterogenous changes as the immune system ages.^109^ This process is characterized by a decline in naïve T cells but an increase in highly differentiated CD28^−^ memory T cells, a phenomenon known as naïve‐memory ratio imbalance. T cell replenishment mainly relies on thymus output. Thereby, thymus degeneration reduces the output of naïve T cells and destroys T cell homeostasis, which explains the age‐related failure of adaptive immunity.^110^, ^111^ Conversely, memory T cells gradually accumulate with ageing.^112^, ^113^, ^114^ Emerging evidence indicated that CD4^+^ T cells could adapt to the challenges of senescence and maintain a slight balance between naïve and memory cells, while CD8^+^ T cells exhibit a significant imbalance in this regard.^115^ In healthy elderly individuals, a decrease in circulating naïve CD8^+^ T cells is a crucial and consistently noted sign of immunosenescence.

Senescent T cells lose the capacity for antigen‐specific killing. In the TME, T‐cell activation needs TCR (signal‐1) and costimulatory signaling (signal‐2) to be present simultaneously. However, important components of TCR signaling (CD3) and costimulatory molecules (CD27 and CD28) are decreased in senescent T cells.^105^, ^116^ Moreover, the diversity of TCR declines with age, particularly in senescent CD8^+^ T cells.^117^, ^118^ Senescent T cells display decreased expression of functional molecules, such as perforin, granzyme B, and SA‐β‐gal, leading to a weakened antitumor activity in mice or patients.^119^, ^120^, ^121^ Senescent T cells develop a hypersecretory, proinflammatory phenotype to remodel the TME. SASP is both a consequence of cellular senescence and a driver for further senescence.^122^ On the one hand, SASP factors, like IL‐6 and TNF‐α, accelerate T cell senescence in an autocrine or paracrine manner.^123^, ^124^ On the other hand, senescent T cells secrete SASP factors leading to the TME with features of an “immune desert,” also known as an immune cold microenvironment, refers to a specific state of tumors characterized by a limited immune response.^125^, ^126^, ^127^ The CCL5/CCR5 axis facilitates the induction and recruitment of M_2_‐type macrophages and MDSCs that impede effector T cell function.^128^ CXCL1 not only prompts adjacent cell senescence but also induces immune evasion via paracrine signaling.^51^ IL‐6 also recruits MSDCs into the TME and drives EMT, triggering tumor growth and invasiveness.^61^, ^86^, ^129^ Also, it has been proven that IL‐18 increases the generation of MDSCs and augments their immunosuppressive activity in multiple myeloma.^130^ A high level of IFN‐γ induced by senescent T cells, could inhibit T cell cytotoxicity by upregulating several immunosuppressive factors, such as IDO, PD‐L1, and CTLA‐4.^131^ Therefore, SASPs reshape the TIME via regulating immunosuppressive cell infiltration. In addition to remodeling the TIME by SASP factors, Treg cells, a unique subpopulation of T cells, directly impede effector T cell function. Given that the change of Treg cells in age‐related pathology is hotly debated, Treg cells may play a context‐specific role in different tumors.^132^, ^133^ Focusing on T‐cell senescence, stress facilitates a general dysfunction of ETC (electron transport chain) components and OXPHOS (oxidative phosphorylation) subunits, leading to elevated ROS generation, dysfunctional mitochondria synthesis, and impaired one‐carbon metabolism.^82^, ^106^, ^134^ Moreover, excessive glucose consumption by tumor cells inevitably results in responder T‐cell senescence, a new strategy for tumor immune evasion.^104^ Overall, the accumulation of senescent T cells in the TME contributes to immune evasion and tumor progression.

##### Monocytes/macrophages

Senescent macrophages, major contributors to immunosenescence, present deterioration in phagocytic capacity and defects in the ability to fight external threats.^135^, ^136^ A large amount of evidence indicates that the immunosuppressive M_2_ tumor‐associated macrophages display a high infiltration in the spleen, bone marrow, and lymphoid tissues of aged mice, facilitating tumor progression in an ageing context.^137^, ^138^, ^139^ Furthermore, macrophages from old individuals exhibit weakened antigen‐presenting ability attributed to declined expression of MHC‐II and coreceptors.^140^, ^141^, ^142^ Significant downregulation of glycolysis and mitochondrial OXPHOS has been demonstrated in aged macrophages, giving rise to an energy‐depleted state that impedes normal macrophage function.^143^

##### Neutrophils

Neutrophils undergo immunosenescence in a low‐grade inflammatory environment, with deterioration in their metabolism and immune function that prompt tumor progression.^144^, ^145^ The age‐related neutrophils exhibit a decline in phagocytic capacity, abnormalities in adhesion, chemotaxis trap network release, increased apoptosis, and aberrant Toll‐like receptor function.^146^, ^147^, ^148^, ^149^, ^150^, ^151^ N2‐type tumor‐associated neutrophils (TAN) represent a distinct subpopulation that has been described in various types of tumors.^152^, ^153^ Studies have shown that the infiltration level of N2‐TANs in the TME is higher in older mice than in young mice. And these suppressive N2‐TANs share similar functions with MDSCs.^154^ However, the direct involvement of senescence‐induced N_2_‐TAN in the TIME still needs further investigation.

##### MDSCs

MDSCs are a population of immunosuppressive cells that accumulate in patients with cancer and establish a premetastatic niche in distant organs.^155^ MDSCs hamper immune surveillance by impairing the function of NK and CD8^+^ T cells, both affecting innate and adaptive immunity.^129^, ^156^ Young mice implanted with breast cancer cells present an increased infiltration of effector T cells, whereas aged mice exhibit a consistent increase in MDSC number.^157^, ^158^ In senescent MDSCs, upregulated expression of the chemokine receptor CX3R1 induced by p16 and p21 overexpression could enhance MDSC recruitment into tumor sites to mediate a protumorigenic effect.^159^

##### NK cells

NK cells are basic components of innate immunity and act as the first defense mechanism in humans.^160^ The ageing process leads to an elevated number of NK cells but compromises their ability to kill targets.^161^, ^162^ Specifically, age‐related NK cells exhibit reduced effector function, with reduced secretion of perforin, granzyme, and IFN‐γ.^163^, ^164^

### 4.2 Tumor cell senescence triggering a switch from immune evasion to immune elimination

**FIGURE 4:**
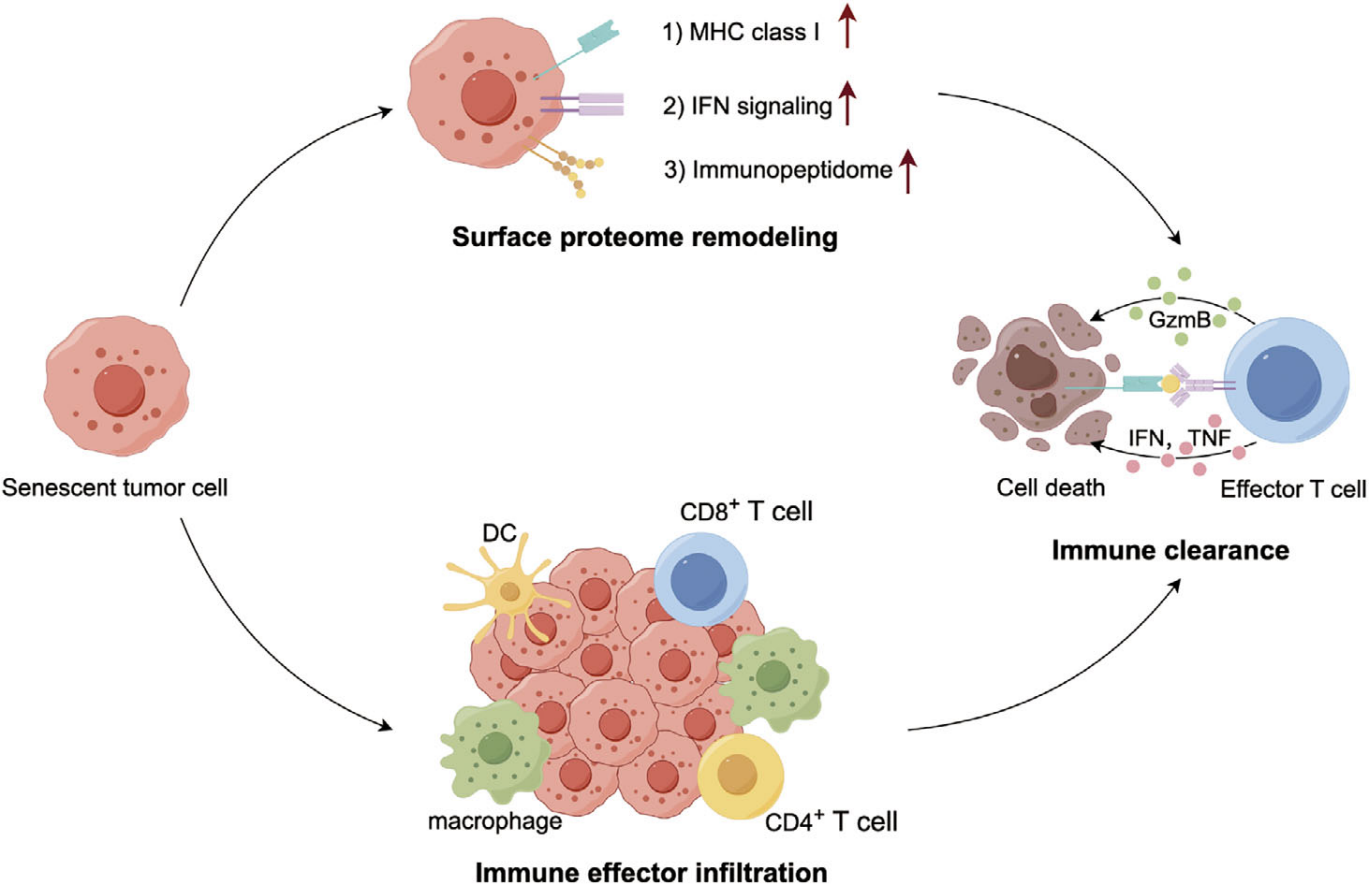
Senescent tumor cells regulate the antitumor immune response. Senescent tumor cells prompt a switch from immune evasion to immune clearance by remodeling their surface proteome, which leads to the following consequences: (1) enhanced IFN‐γ signaling, (2) upregulated MHC‐I levels, and (3) altered immunopeptidome expression. Meanwhile, this robust antigen presentation machinery increases the engagement of effector immune cells, such as effector T cells, dendritic cells, and macrophages. These cells work together to clear tumor cells from the host. IFN‐γ, interferon‐γ; MHC‐I, major histocompatibility complex‐I.

Cellular senescence is a damage‐induced response characterized by a stable cell cycle arrest and a secretory program. In some contexts, senescence serves as an endogenous homeostatic mechanism to drive the immune clearance of senescent cells (Figure 4).^165^

As discussed above, senescence‐associated immune surveillance exerts potent antitumor roles.^71^, ^79^ Importantly, the molecular basis by which senescent tumor cells become visible to the immune system will facilitate novel treatment strategies to boost antitumor immunity.^166^, ^167^ On the one hand, tumor cell senescence directs an abrupt switch from immune evasion to immune elimination mediated by TIME remodeling, laying the basis for productive antitumor immunity. In an immune‐competent liver cancer model, p53‐driven tumor senescence moderately declined the percentage of immune suppressors (MDSCs and neutrophils) while prominently increasing the infiltration of immune effectors (macrophages, CD4^+^ T cells, and CD8^+^ T cells), driving a shift from an immune‐incompetent to an immune‐competent TME.^166^ On the other hand, senescence reshapes the cell‐surface proteome to rewire how tumor cells sense microenvironment signals.^166^, ^167^ First, senescent cells augment the IFN‐γ receptor IFNGR1 and become hypersensitive to microenvironmental IFN‐γ signaling.^166^ Secon, senescent cells upregulate MHC class I expression to enhance their antigen‐presenting capacity, which can be explained by their hyperactivated IFN‐γ signaling.^167^ Senescent cells present an enhanced capacity to send and receive environmental signals necessary for effective adaptive immunity. Destroying the integrity of IFN‐γ signaling in senescent cells will blunt their immune‐dependent clearance. Additionally, senescent cells generate a secretome containing immune‐modulatory factors, increase antigen presentation, and express costimulatory receptors such as CD137L and OX40L to facilitate T cell activation.^168^

The TME remodeling and sensing programs engaged by senescence allow tumor cells to be more visible to immune surveillance. These immediate‐early senescent tumor cell responses facilitate immune‐mediated tumor cell eradication. However, in the late stages of senescence induction, senescent tumor cells may express immune checkpoints that engage coinhibitory receptors on immune cells.^169^, ^170^ Overall, senescent cells have an immunogenic potential that can be harnessed to prime the immune system toward tumor clearance but needs to be carefully wielded to avoid their immunosuppressive effects.^167^, ^168^

## 5 TARGETING SENESCENCE FOR ANTITUMOR THERAPY

Activation of the host immune system is considered an attractive way to eliminate senescent cancer cells.^171^ Existing drugs targeting senescence that selectively eliminate senescent cells (senolytics) or inhibit the SASP (senomorphics) are promising anticancer therapies. Alternatively, therapeutic strategies for reversing senescence of tumor‐specific T cells have emerged to enhance cancer immunotherapy. Potential interventions targeting senescence are summarized in Table 2.

**TABLE 2:** Targeting senescence for antitumor therapy.

Intervention	Type	Example	References
Senolytic therapy	BCL‐2 family inhibitor	Navitoclax, ABT‐737, A1155463, A1331852, EF24, venetoclax	^172^, ^173^, ^174^
	mTOR inhibitor	AZD8055, temsirolimus, sertraline	^175^, ^176^, ^177^
	p53 activity modulator	FOXO4‐DRI peptide, UBX0101	^178^, ^179^
	BET family degrader	ARV825	^180^, ^181^
	Cardiac glycosides	Digoxin, ouabain	^182^, ^183^
	Immunotherapy	PD1/PD‐L1 blocking antibodies, CAR‐T cells	^184^, ^185^, ^186^
Senomorphic therapy	SASP regulator	Metformin, Rapamycin, ruxolitinib	^187^, ^188^, ^189^
	SASP antibody	Siltuximab, canakinumab	^190^, ^191^
	Hormone	Melatonin, androgens, estrogens, estradiol, glucocorticoids	^192^, ^193^, ^194^, ^195^
Senescent T cells	Metabolic regulator	Rapamycin, metformin, BIRB 796, HBOT, 7‐ddA, H89, ssRNA40	^103^, ^125^, ^196^, ^197^, ^198^, ^199^, ^200^
	Signaling pathway regulator	MAPK inhibitors, ATM inhibitors	^201^, ^202^, ^203^

### 5.1 Modulating senescence‐related tumor cells and SASPs

#### 5.1.1 Senolytic therapies

The persistence of therapy‐induced senescent cells can lead to adverse outcomes, including an increased incidence of secondary tumors, the recurrence of more aggressive cells, and complications related to treatment.^204^, ^205^ This argues for a strategy to eliminate senescent tumor cells by senolytic agents, which can selectively induce apoptosis in senescent cells but spare other nonsenescent cells.^206^ Notably, a key feature of senescent cells is a change in chromatin structure that alters gene expression. These changes can affect crucial processes like apoptosis regulation, creating new vulnerabilities in senescent cells that can be specifically targeted by senolytic drugs.^8^ Therefore, applying senolytic agents in combination with or following senescence‐inducing therapy may generate unexpected clinical benefits.

BCL‐X_L_‐modulating drugs have shown the greatest promise in obliterating senescent tumor cells by targeting antiapoptotic BCL‐2 pathways.^207^ For example, navitoclax (ABT‐263), selectively targeting BCL‐2, BCL‐X_L_, and BCL‐W proteins, could effectively eliminate senescent cells by inducing programmed cell death.^172^, ^208^ As reported, navitoclax could kill breast and ovarian cancer cells after application of PARP inhibitors in aged animal models and in vitro culture.^209^ Exposure to navitoclax following etoposide or doxorubicin showed strong effectiveness in slowing tumor progression.^204^, ^210^ Consistently, in a phase‐II study, the combination of navitoclax and rituximab showed higher effectiveness than rituximab monotherapy accompanied by well tolerance in patients with chronic lymphocytic leukemia.^211^ Other BCL‐2 family inhibitors, such as ABT‐737, A1155463, A1331852, and EF24, have been developed as promising senolytic drugs.^165^, ^173^, ^174^

However, the side effects of navitoclax primarily arise from its off‐target effects on hematological cells, including thrombocytopenia and neutropenia, which significantly limits its broader use.^212^ Several attempts have been made to address this challenge. Galacto‐conjugated navitoclax prodrug (nav‐gal) has been designed to improve delivery accuracy.^213^ This prodrug is processed by SA‐β‐gal in senescent cells, thereby specifically killing senescent cells and limiting off‐target effects in normal cells. The combination of nav‐gal and cisplatin has demonstrated efficient clearance of lung cancer cells. Moreover, nanocarrier‐encapsulated doxorubicin is another strategy to induce specific cytotoxicity in senescent cells, which has been validated in various studies.^214^, ^215^ Alternatively, the proteolysis‐targeting chimera (PROTAC) drug PZ15227 is reported to limit platelet toxicity of navitoclax by hijacking the cereblon (CRBN) E3 ligase for BCL‐XL degradation. Given the minimal expression of CRBN in platelets, PZ15227 could eliminate senescent tumor cells without inducing severe thrombocytopenia.^216^

The mTOR inhibitor AZD8055 also showed potent senolytic effects against cancer cells with a senescent phenotype induced by DNA‐replication kinase CDC7 inhibitors.^177^, ^217^ Similarly, the dual treatment of docetaxel and mTOR inhibitor temsirolimus results in a remarkable reduction of tumor growth in prostate and breast cancer animal models.^175^ Sertraline, a selective serotonin reuptake inhibitor, is frequently used to treat psychological disorders. By suppressing mTOR signaling, sertraline was identified as a senolytic agent to kill hepatocellular carcinoma cells that have been rendered senescent through CDC7 inhibition.^177^ Nevertheless, the senolytic effect of mTOR inhibitors still requires further exploitation and tests in more tumor types.

Moreover, pharmacological interventions that regulate p53 activity have senolytic effects. Given that FOXO4 can retain p53 in the nucleus, the FOXO4‐DRI peptide could disrupt the p53–FOXO4 interaction, leading to p53‐mediated apoptosis in senescent cells.^178^ Despite the FOXO4‐DRI peptide displaying well tolerance in mice, its clinical use remains lacking enough evaluation. Notably, compounds that modulate p53 activity are only effective in tumors with p53‐wild‐type, significantly limiting their therapeutic scope.^218^, ^219^

The BET family protein degrader ARV825 provokes senolysis via attenuating nonhomologous end‐joining (NHEJ) DNA DSB repair and upregulating autophagic gene expression.^180^, ^181^ In mice undergoing doxorubicin‐induced senescence, ARV825 treatment effectively eliminates senescent hepatic stellate cells, leading to delayed development of liver cancer.^181^

Cardiac glycosides, including widely used digoxin, could selectively kill senescent cells in multiple cancer models.^182^, ^183^ Cardiac glycosides that inhibit the Na^+^/K^+^ ATPase pump could drive cell depolarization and acidification, ultimately leading to senescent cell death.^183^ Besides, these compounds can activate the proapoptotic BCL‐2 family protein NOXA to trigger intrinsic apoptosis.^182^

Although numerous small molecules have demonstrated encouraging efficacy against cellular senescence, these agents exhibit a deficiency in sensitivity and may result in significant side effects. During the past decades, immunotherapy has gained breakthroughs in treating patients with advanced or drug‐resistant malignancies.^220^ Immunotherapy could mediate endogenous senolysis to prompt senescent cell eradication. Antiprogrammed cell death protein 1 (PD1) blockades can elicit senolytic effects following TIS. The combination of trametinib and palbociclib induced senescence in a mouse model of *KRAS*‐mutant PDAC, accompanied by increased tumor vascularization and endothelial cell activation, ultimately improving the efficacy of anti‐PD1 therapy.^68^ Notably, chimeric antigen receptor T‐cell (CAR‐T) therapy directed at senescence‐specific surface antigens represent a viable treatment strategy, ablating senescent cells in vitro and in vivo. The urokinase‐type plasminogen activator receptor (uPAR) as a cell membrane protein is significantly upregulated following senescence induction. uPAR‐specific CAR‐T cells extended the tumor growth in mice with lung adenocarcinoma previously treated with a combination of MEK and CDK4/6 inhibitors.^184^ Other cell surface proteins might be potential targets for cancer immunotherapy, such as DEP1, DPP4, NKG2D, ICAM1, NOTCH1, and NOTCH3.^221^, ^222^, ^223^, ^224^, ^225^, ^226^ Given these promising findings, more clinical studies are required to explore whether the benefits of senolytics outweigh their potential side events and to determine the optimal dose schedule. We have summarized relevant clinical trials in Table 3.

**TABLE 3:** Clinical trials targeting senescence‐related biomarkers.

Targeted tumor type	Agents	Targets	Identifier	Phase	Enrollment
B‐cell CLL	ABT‐263	BCL‐2, BCL‐XL	NCT01087151	Phase‐2	118
DLBCL	Temsirolimus	mTOR	NCT01653067	Phase‐2	88
Breast cancer	Anti‐PD‐L1	PD‐L1	NCT04360941	Phase‐1	45
Advanced solid tumors	Sym023	Tim‐3	NCT03489343	Phase‐1	24
Liver cancer	TSR‐022, TSR‐042	Tim‐3, PD‐1	NCT03680508	Phase‐2	42
Melanoma	TSR‐022, TSR‐042	Tim‐3, PD‐1	NCT04139902	Phase‐2	56
Cervical cancer	BGB‐A317, BGB‐A1217	TIGIT, PD‐1	NCT04693234	Phase‐2	178
ESCC	BGB‐A317, BGB‐A1217	TIGIT, PD‐1	NCT04732494	Phase‐2	125
Multiple myeloma	CD3/CD28	KLRG‐1	NCT01426828	Phase‐2	40
HIV‐associated HL	Ipilimumab, nivolumab	PD‐1, CTLA‐4	NCT02408861	Phase‐1	96

Abbreviations: CLL, chronic lymphocytic leukemia; DLBCL, diffuse large B‐cell lymphoma; ESCC, esophageal squamous cell carcinoma; HL, Hodgkin lymphoma (https://clinicaltrials.gov/).

#### 5.1.2 Senomorphic therapies

Senomorphic SASP inhibitors could function as efficacious alternatives to senolytics. Metformin, a widely recognized medication, could prevent the translocation of NF‐κB pathway components to the nucleus and inhibit their subsequent activation, thus leading to a decrease in the expression of diverse SASP factors. This underlying mechanism could plausibly elucidate the antiaging and antitumor effects of metformin in both murine models and diabetic patients.^187^ mTOR inhibitor, such as rapamycin, could reduce NF‐λB activity, suppress inflammatory SASPs at the translational level, and constrain the tumor‐prompting effect of senescent bystander fibroblasts.^52^, ^188^ Inhibitors of the JAK signaling pathway in aged mice, such as ruxolitinib, could reduce inflammation and ameliorate frailty by suppressing SASP factors. Currently, several JAK inhibitors are being tested in clinical trials including patients with acute myeloid leukemia or lymphomas.^189^, ^227^

Antibodies against SASP factors also have senormophic effects. For example, siltuximab, an antibody inhibiting IL‐6 approved for the treatment of multicentric Castleman disease, has shown activities in diverse oncological contexts.^190^ Canakinumab, an anti‐IL‐1β monoclonal antibody approved for pyrexia‐featured inflammatory syndromes, is evaluated in clinical trials involving patients diagnosed with non‐small cell lung cancer.^191^

Some hormones may hold the potential to limit detrimental SASP factors. As a novel SASP suppressor, melatonin suppresses SASP gene expression via modulating poly (ADP‐ribose) polymerase 1 (PARP1).^195^ Other hormones, such as androgens, estrogens, and glucocorticoids, also possess the ability to regulate the secretion of proinflammatory SASP factors.^192^, ^193^, ^194^ Notably, further investigation is necessary to thoroughly examine the anticancer efficacy of these senescence‐dependent, SASP‐suppressing agents in suitable model systems and clinical trials.

### 5.2 Therapeutic strategies for preventing T‐cell senescence

The failure of immunity to eradicate tumor cells is mainly attributed to T‐cell dysfunction, with flaws in proliferation and effector function. Increasing evidence has shed light on T‐cell senescence used by malignant cells to achieve immune evasion. Therefore, novel strategies to prevent the occurrence of senescence and control the fate of tumor‐specific T cells hold the potential to hinder tumor development and optimize cancer immunotherapy.

#### 5.2.1 Targeting metabolic reprogramming

Senescence is the culmination of the common effect of internal and external stimuli. Metabolic stresses, such as glucose deprivation, mitochondrial dysfunction, imbalanced lipid metabolism, hypoxia, and tumor metabolites, are critical to regulating T‐cell senescence.

Excessive glucose consumption by tumor cells and Treg cells leads to a deficiency of glucose supply for effector T cells, ultimately triggering responder T‐cell senescence.^103^, ^104^ The addition of glucose could avoid effector T‐cell senescence induced by glucose competition.^228^ Molecular inhibitors targeting mTORC1 or p53 hold the potential to rejuvenate mitochondrial fitness, thereby preventing T‐cell senescence.^125^, ^198^, ^199^ There is a mechanistic link between T‐cell senescence and lipid metabolic reprogramming in the TME. Findings identify that senescent T cells display hyperactive glycolysis but imbalanced lipid metabolism.^229^ The latter changes the expression of lipid metabolic enzymes, which, in turn, affects the species and amount of lipid droplets accumulated in T cells.^230^ Specifically, over‐expression of group IVA phospholipase A_2_ in malignant and Treg cells could induce T‐cell senescence. Inhibition of group IVA phospholipase A_2_ activity is reported to alter T‐cell lipid metabolism, prevent T‐cell senescence in vitro, and boost antitumor immunity in in vivo models of melanoma and breast cancer.^231^ The hypoxic TME may also induce T‐cell senescence.^197^ Consequently, hyperbaric oxygen therapy (HBOT) is confirmed to exert senolytic effects by increasing telomere length and reducing immunosenescence in peripheral blood cells.^200^ Alternatively, downregulating tumor‐derived cAMP in the TME by cAMP pharmacological inhibitors or activating the TLR8 signaling pathway by synthetic poly‐G3 and natural TLR8 ligand can prevent the induction of senescence in responder T cells while reversing the suppressive effect of senescent T cells.^103^, ^196^, ^197^ Ample evidence supports that TLRs directly participate in metabolic reprogramming to interfere with malignant behavior in several solid tumors.^197^, ^232^, ^233^, ^234^ Therefore, TLR agonists might be effective agents or adjuvants for cancer immunotherapy.

#### 5.2.2 Manipulating signaling pathways

The importance of the mitogen‐activated protein kinase (MAPK) signaling pathway in mediating T‐cell senescence has recently been exploited.^105^, ^235^ Molecular inhibitors targeting ERK, p38, and STAT signaling pathways may reverse or prevent T‐cell senescence.^90^, ^236^, ^237^, ^238^, ^239^ Meanwhile, the MAPK signaling pathway plays a vital role in T‐cell activation and effector functions.^235^, ^240^ Hence, it is urgently needed to select the optimal MAPK inhibitors for restraining senescence induction without concession in self‐renewal and cytotoxicity of tumor‐reactive T cells.

Selective MAPK inhibitors already available for clinical trials are preferential candidates for treating melanoma patients, with improved T‐cell recognition of tumor cells without affecting lymphocyte function.^201^, ^202^ Moreover, the CRISPR/Cas9 gene editing technology could modulate the MAPK signaling pathway more stably and precisely. Similarly, selective ATM inhibitors have been tested in clinical trials for cancer patients.^203^, ^241^ Furthermore, the role of sestrins in regulating immunosenescence is multifaceted. Increasing evidence supports that sestrins coordinate ERK, JNK, and P38 phosphorylation in CD4^+^ T cells. Sestrin knockdown restores T‐cell responsiveness and cytokine production in vivo during ageing.^242^ Notably, sestrins may induce the programming of senescent CD8^+^ T cells to obtain NK‐like killing ability but lose TCR‐dependent signaling activity.^116^ More mechanisms of sestrin‐mediated T‐cell senescence need further elaboration.

## 6 CHALLENGES AND FUTURE DIRECTIONS

A two‐step therapy, consisting of prosenescence therapy followed by senolytic intervention, seems theoretically sound. This is because senescence is a stable cellular condition that continues even after the inducing factor is eliminated. Therefore, a primary unmet need is the lack of drugs that are highly effective in inducing senescence in a high percentage of cancer cells. Meanwhile, such medications must be highly selective to target cancer cells instead of healthy cells, as triggering senescence in normal tissues can lead to harmful side effects.

Another potential challenge lies in the lack of a reliable quantitative assessment of the unique contribution of each SASP factor and the absence of gold standard biomarkers for detecting the senescence state in clinical settings. Currently, no single indicator could reliably distinguish senescence from other arrested states. Noninvasive imaging methods would be ideal for measuring the effectiveness of senescence induction in tumors of patients on therapy. Besides, liquid biopsy could be used to detect senescent cells. For example, oxylipin production rises significantly during senescence.^243^ The intracellular prostaglandin dihomo‐15D‐PGJ2, a specific type of oxylipin, notably accumulates in senescent cells and is discharged during senolysis. As such, dihomo‐15D‐PGJ2 could be detected in urine and blood samples from patients. Alternatively, noninvasive detection of SASP factors in plasma might uncover senescence burdens.^14^

A further issue is tumor heterogeneity that may limit the efficacy of senescence induction within tumors. Appropriate preclinical models, such as organoids, can better imitate the TME and resemble a more physiological human cancer model. Also, single‐cell and spatial sequencing help to map the location of distinct cell types and subpopulations in the TME, thereby clarifying the complicated interplay of cellular senescence, immunity, and tumor evolution. Additionally, senescent cells can disseminate the senescence phenotype through the SASP to the adjacent nonsenescent cells within tumors; however, this has yet to be substantiated. These bystander effects in the context of senescence‐inducing therapies hold the potential to overcome tumor heterogeneity related to treatment responsiveness.

Using senolytic therapies in the old people requires careful assessments. Clearly, senescent cells constitute a large percentage of the number of cells, which could compromise tissue structural integrity or impact vascular endothelial cells, resulting in blood–tissue barrier dysfunction. Therefore, this issue highlights the urgent need to develop tumor cell‐specific senolytic agents.

Overall, although numerous open questions still linger unresolved, significant benefits of senescence‐based therapies warrant further investigation in this field.

## 7 CONCLUSION

Cellular senescence has traditionally been regarded as a tumor‐suppressive mechanism whereby the proliferation of dysfunctional cells susceptible to malignant transformation is halted. However, a more intricate perspective has risen regarding the involvement of cellular senescence in tumorigenesis and response to cancer therapy over the past decades. Given the highly complicated and context‐dependent effects of cellular senescence in cancer, weighing the balance of its “bright” and “dark” sides is inevitably critical. Similarly, the intersection of immune surveillance and tumor evolution harbors considerable complexities as well. Increasing evidence suggests the TME drives immunosenescence through multiple pathways. The accumulation of senescent immune cells potentially contributes to progressive tumors and limits the efficacy of cancer treatment. More importantly, senescent tumor cells possess an immunological capacity that can be harnessed to provoke immune surveillance. Of note, wielding this potential should be cautious to mitigate the immunosuppressive, protumorigenic capacities of senescent cells. From the perspective of translational medicine, a one‐two‐punch sequential therapy is feasible, whereby tumor cells are treated with senescence‐induction therapy followed by senolytics or senomorphics. Activating the host immune system presents a highly appealing way to eliminate senescent cells. Preventing T‐cell senescence might be a novel strategy to enhance cancer immunotherapy. Future studies should continue to conduct research on senescence and immunotherapy synergies. Therefore, exploiting cellular senescence as an antitumor therapeutic strategy will hold great interest in the future.

## AUTHOR CONTRIBUTIONS

Zehua Wang contributed to study design, performed literature search, and drafted the original manuscript. Chen Chen contributed to study design, performed literature search, and confirmed the final manuscript. Jiaoyu Ai, Yaping Gao, Lei Wang and Shurui Xia critically revised of the manuscript for important intellectual content. Yongxu Jia and Yanru Qin critically supervised the whole study as the expert and confirmed the final manuscript. All authors read and approved the final manuscript.

## CONFLICT OF INTEREST STATEMENT

The authors declare that they have no conflicts of interest.

## ETHICS STATEMENT

Not applicable.

## Data Availability

Not applicable.

## References

[1] Hernandez‐Segura A , Nehme J , Demaria M . Hallmarks of cellular senescence. Trends Cell Biol. 2018;28(6):436‐453. 29477613 10.1016/j.tcb.2018.02.001

[2] Hayflick L , Moorhead PS . The serial cultivation of human diploid cell strains. Exp Cell Res. 1961;25:585‐621. 13905658 10.1016/0014-4827(61)90192-6

[3] Campisi J . Aging, cellular senescence, and cancer. Annu Rev Physiol. 2013;75:685‐705. 23140366 10.1146/annurev-physiol-030212-183653 PMC4166529

[4] Demaria M , Ohtani N , Youssef SA , et al. An essential role for senescent cells in optimal wound healing through secretion of PDGF‐AA. Dev Cell. 2014;31(6):722‐733. 25499914 10.1016/j.devcel.2014.11.012 PMC4349629

[5] Jun JI , Lau LF . The matricellular protein CCN1 induces fibroblast senescence and restricts fibrosis in cutaneous wound healing. Nat Cell Biol. 2010;12(7):676‐685. 20526329 10.1038/ncb2070 PMC2919364

[6] Storer M , Mas A , Robert‐Moreno A , et al. Senescence is a developmental mechanism that contributes to embryonic growth and patterning. Cell. 2013;155(5):1119‐1130. 24238961 10.1016/j.cell.2013.10.041

[7] Deng Y , Chang S . Role of telomeres and telomerase in genomic instability, senescence and cancer. Lab Invest. 2007;87(11):1071‐1076. 17767195 10.1038/labinvest.3700673

[8] Ewald JA , Desotelle JA , Wilding G , Jarrard DF . Therapy‐induced senescence in cancer. J Natl Cancer Inst. 2010;102(20):1536‐1546. 20858887 10.1093/jnci/djq364 PMC2957429

[9] Gorgoulis V , Adams PD , Alimonti A , et al. Cellular senescence: defining a path forward. Cell. 2019;179(4):813‐827. 31675495 10.1016/j.cell.2019.10.005

[10] Kuilman T , Michaloglou C , Mooi WJ , Peeper DS . The essence of senescence. Genes Dev. 2010;24(22):2463‐2479. 21078816 10.1101/gad.1971610 PMC2975923

[11] Hanahan D . Hallmarks of Cancer: New Dimensions. Cancer Discov. 2022;12(1):31‐46. 35022204 10.1158/2159-8290.CD-21-1059

[12] Campisi J , d'Adda di Fagagna F . Cellular senescence: when bad things happen to good cells. Nat Rev Mol Cell Biol. 2007;8(9):729‐740. 17667954 10.1038/nrm2233

[13] Tomaselli D , Steegborn C , Mai A , Rotili D . Sirt4: A Multifaceted Enzyme at the Crossroads of Mitochondrial Metabolism and Cancer. Front Oncol. 2020;10:474. 32373514 10.3389/fonc.2020.00474 PMC7177044

[14] Basisty N , Kale A , Jeon OH , et al. A proteomic atlas of senescence‐associated secretomes for aging biomarker development. PLoS Biol. 2020;18(1):e3000599. 31945054 10.1371/journal.pbio.3000599 PMC6964821

[15] Coppé JP , Patil CK , Rodier F , et al. Senescence‐associated secretory phenotypes reveal cell‐nonautonomous functions of oncogenic RAS and the p53 tumor suppressor. PLoS Biol. 2008;6(12):2853‐2868. 19053174 10.1371/journal.pbio.0060301 PMC2592359

[16] Xie Y , Xie F , Zhang L , et al. Targeted anti‐tumor immunotherapy using tumor infiltrating cells. Advanced science (Weinheim, Baden‐Wurttemberg, Germany). 2021;8(22):e2101672. 34658167 10.1002/advs.202101672 PMC8596143

[17] Yang Y , Fan D , Zheng BH , Zhou ST . [Latest findings on the function of immune metabolism in tumor immunity]. Sichuan da xue xue bao Yi xue ban = Journal of Sichuan University Medical science edition. 2023;54(3):497‐504. 37248574 10.12182/20230560304 PMC10475430

[18] Walford RL . The immunologic theory of aging. Gerontologist. 1964;4:195‐197. 14289265 10.1093/geront/4.4.195

[19] Pawelec G . Immunosenescence comes of age. Symposium on aging research in immunology: the impact of genomics. EMBO Rep. 2007;8(3):220‐223. 17304236 10.1038/sj.embor.7400922 PMC1808042

[20] Pawelec G . Age and immunity: What is “immunosenescence”? Exp Gerontol. 2018;105:4‐9. 29111233 10.1016/j.exger.2017.10.024

[21] Hanna A , Balko JM . No rest for the wicked: Tumor cell senescence reshapes the immune microenvironment. Cancer Cell. 2023;41(5):831‐833. 37059102 10.1016/j.ccell.2023.03.013

[22] Kudlova N , De Sanctis JB , Hajduch M . Cellular senescence: molecular targets, biomarkers, and senolytic drugs. Int J Mol Sci. 2022;23(8):4168. 35456986 10.3390/ijms23084168 PMC9028163

[23] Schmitt CA , Wang B , Demaria M . Senescence and cancer ‐ role and therapeutic opportunities. Nat Rev Clin Oncol. 2022;19(10):619‐636. 36045302 10.1038/s41571-022-00668-4 PMC9428886

[24] Wang B , Han J , Elisseeff JH , Demaria M . The senescence‐associated secretory phenotype and its physiological and pathological implications. Nat Rev Mol Cell Biol. 2024;25(12):958‐978. 38654098 10.1038/s41580-024-00727-x

[25] von Zglinicki T , Wan T , Miwa S . Senescence in post‐mitotic cells: a driver of aging? Antioxid Redox Signaling. 2021;34(4):308‐323. 10.1089/ars.2020.8048 PMC7821432 32164429

[26] Sanokawa‐Akakura R , Akakura S , Ostrakhovitch EA , Tabibzadeh S . Replicative senescence is distinguishable from DNA damage‐induced senescence by increased methylation of promoter of rDNA and reduced expression of rRNA. Mech Ageing Dev. 2019;183:111149. 31568766 10.1016/j.mad.2019.111149

[27] Pizzul P , Rinaldi C , Bonetti D . The multistep path to replicative senescence onset: zooming on triggering and inhibitory events at telomeric DNA. Front Cell Dev Biol. 2023;11:1250264. 37771378 10.3389/fcell.2023.1250264 PMC10524272

[28] Alcorta DA , Xiong Y , Phelps D , Hannon G , Beach D , Barrett JC . Involvement of the cyclin‐dependent kinase inhibitor p16 (INK4a) in replicative senescence of normal human fibroblasts. Proc Nat Acad Sci USA. 1996;93(24):13742‐13747. 8943005 10.1073/pnas.93.24.13742 PMC19411

[29] Herr LM , Schaffer ED , Fuchs KF , Datta A , Brosh RM, Jr . Replication stress as a driver of cellular senescence and aging. Commun Biol. 2024;7(1):616. 38777831 10.1038/s42003-024-06263-w PMC11111458

[30] Zhou S , Cui J , Shi Y . Serine metabolism regulates the replicative senescence of human dental pulp cells through histone methylation. Curr Issues Mol Biol. 2024;46(4):2856‐2870. 38666909 10.3390/cimb46040179 PMC11049641

[31] Wu F , Zhang L , Lai C , et al. Dynamic alteration profile and new role of RNA m6A methylation in replicative and H(2)O(2)‐induced premature senescence of human embryonic lung fibroblasts. Int J Mol Sci. 2022;23(16):9271. 36012545 10.3390/ijms23169271 PMC9408987

[32] Cao L , Lee SG , Park SH , Kim HR . Sargahydroquinoic acid (SHQA) suppresses cellular senescence through Akt/mTOR signaling pathway. Exp Gerontol. 2021;151:111406. 34022274 10.1016/j.exger.2021.111406

[33] Dasi D , Nallabelli N , Devalaraju R , et al. Curcumin attenuates replicative senescence in human dental follicle cells and restores their osteogenic differentiation. Journal of oral biosciences. 2023;65(4):371‐378. 37806337 10.1016/j.job.2023.10.001

[34] Braig M , Lee S , Loddenkemper C , et al. Oncogene‐induced senescence as an initial barrier in lymphoma development. Nature. 2005;436(7051):660‐665. 16079837 10.1038/nature03841

[35] Muñoz‐Espín D , Serrano M . Cellular senescence: from physiology to pathology. Nat Rev Mol Cell Biol. 2014;15(7):482‐496. 24954210 10.1038/nrm3823

[36] Sharpless NE , Sherr CJ . Forging a signature of in vivo senescence. Nat Rev Cancer. 2015;15(7):397‐408. 26105537 10.1038/nrc3960

[37] Chen Z , Trotman LC , Shaffer D , et al. Crucial role of p53‐dependent cellular senescence in suppression of Pten‐deficient tumorigenesis. Nature. 2005;436(7051):725‐730. 16079851 10.1038/nature03918 PMC1939938

[38] Lazzerini Denchi E , Attwooll C , Pasini D , Helin K . Deregulated E2F activity induces hyperplasia and senescence‐like features in the mouse pituitary gland. Mol Cell Biol. 2005;25(7):2660‐2672. 15767672 10.1128/MCB.25.7.2660-2672.2005 PMC1061636

[39] Michaloglou C , Vredeveld LC , Soengas MS , et al. BRAFE600‐associated senescence‐like cell cycle arrest of human naevi. Nature. 2005;436(7051):720‐724. 16079850 10.1038/nature03890

[40] Vickridge E , Faraco CCF , Tehrani PS , et al. The DNA repair function of BCL11A suppresses senescence and promotes continued proliferation of triple‐negative breast cancer cells. NAR cancer. 2022;4(4):zcac028. 36186110 10.1093/narcan/zcac028 PMC9516615

[41] Giacinti C , Giordano A . RB and cell cycle progression. Oncogene. 2006;25(38):5220‐5227. 16936740 10.1038/sj.onc.1209615

[42] Paez‐Ribes M , González‐Gualda E , Doherty GJ , Muñoz‐Espín D . Targeting senescent cells in translational medicine. EMBO Mol Med. 2019;11(12):e10234. 31746100 10.15252/emmm.201810234 PMC6895604

[43] Di Micco R , Fumagalli M , Cicalese A , et al. Oncogene‐induced senescence is a DNA damage response triggered by DNA hyper‐replication. Nature. 2006;444(7119):638‐642. 17136094 10.1038/nature05327

[44] Prasanna PG , Citrin DE , Hildesheim J , et al. Therapy‐induced senescence: opportunities to improve anticancer therapy. J Natl Cancer Inst. 2021;113(10):1285‐1298. 33792717 10.1093/jnci/djab064 PMC8486333

[45] Özdemir A , Şimay Demir YD , Yeşilyurt ZE , Ark M . Senescent cells and SASP in cancer microenvironment: new approaches in cancer therapy. Advances in protein chemistry and structural biology. 2023;133:115‐158. 36707199 10.1016/bs.apcsb.2022.10.002

[46] Murray D , Mirzayans R . Cellular responses to platinum‐based anticancer drugs and UVC: role of p53 and implications for cancer therapy. Int J Mol Sci. 2020;21(16):5766. 32796711 10.3390/ijms21165766 PMC7461110

[47] Bousset L , Gil J . Targeting senescence as an anticancer therapy. Molecular oncology. 2022;16(21):3855‐3880. 36065138 10.1002/1878-0261.13312 PMC9627790

[48] Go S , Kang M , Kwon SP , Jung M , Jeon OH , Kim BS . The senolytic drug JQ1 removes senescent cells via ferroptosis. Tissue engineering and regenerative medicine. 2021;18(5):841‐850. 34003467 10.1007/s13770-021-00346-z PMC8440740

[49] Ouvrier B , Ismael S , Bix GJ . Senescence and SASP are potential therapeutic targets for ischemic stroke. Pharmaceuticals (Basel, Switzerland). 2024;17(3):312. 38543098 10.3390/ph17030312 PMC10973994

[50] Özcan S , Alessio N , Acar MB , et al. Unbiased analysis of senescence associated secretory phenotype (SASP) to identify common components following different genotoxic stresses. Aging. 2016;8(7):1316‐1329. 27288264 10.18632/aging.100971 PMC4993333

[51] Acosta JC , Banito A , Wuestefeld T , et al. A complex secretory program orchestrated by the inflammasome controls paracrine senescence. Nat Cell Biol. 2013;15(8):978‐990. 23770676 10.1038/ncb2784 PMC3732483

[52] Laberge RM , Sun Y , Orjalo AV , et al. MTOR regulates the pro‐tumorigenic senescence‐associated secretory phenotype by promoting IL1A translation. Nat Cell Biol. 2015;17(8):1049‐1061. 26147250 10.1038/ncb3195 PMC4691706

[53] Ritschka B , Storer M , Mas A , et al. The senescence‐associated secretory phenotype induces cellular plasticity and tissue regeneration. Genes Dev. 2017;31(2):172‐183. 28143833 10.1101/gad.290635.116 PMC5322731

[54] Eggert T , Wolter K , Ji J , et al. Distinct functions of senescence‐associated immune responses in liver tumor surveillance and tumor progression. Cancer Cell. 2016;30(4):533‐547. 27728804 10.1016/j.ccell.2016.09.003 PMC7789819

[55] Kang TW , Yevsa T , Woller N , et al. Senescence surveillance of pre‐malignant hepatocytes limits liver cancer development. Nature. 2011;479(7374):547‐551. 22080947 10.1038/nature10599

[56] Lasry A , Ben‐Neriah Y . Senescence‐associated inflammatory responses: aging and cancer perspectives. Trends Immunol. 2015;36(4):217‐228. 25801910 10.1016/j.it.2015.02.009

[57] Kansara M , Leong HS , Lin DM , et al. Immune response to RB1‐regulated senescence limits radiation‐induced osteosarcoma formation. J Clin Invest. 2013;123(12):5351‐5560. 24231354 10.1172/JCI70559 PMC3859382

[58] Vilgelm AE , Johnson CA , Prasad N , et al. Connecting the dots: therapy‐induced senescence and a tumor‐suppressive immune microenvironment. J Natl Cancer Inst. 2016;108(6):djv406. 26719346 10.1093/jnci/djv406 PMC4849355

[59] Loo TM , Kamachi F , Watanabe Y , et al. Gut microbiota promotes obesity‐associated liver cancer through PGE(2)‐mediated suppression of antitumor immunity. Cancer Discov. 2017;7(5):522‐538. 28202625 10.1158/2159-8290.CD-16-0932

[60] Liu D , Hornsby PJ . Senescent human fibroblasts increase the early growth of xenograft tumors via matrix metalloproteinase secretion. Cancer Res. 2007;67(7):3117‐3126. 17409418 10.1158/0008-5472.CAN-06-3452

[61] Di Mitri D , Toso A , Chen JJ , et al. Tumour‐infiltrating Gr‐1+ myeloid cells antagonize senescence in cancer. Nature. 2014;515(7525):134‐137. 25156255 10.1038/nature13638

[62] Kim YH , Choi YW , Lee J , Soh EY , Kim JH , Park TJ . Senescent tumor cells lead the collective invasion in thyroid cancer. Nat Commun. 2017;8:15208. 28489070 10.1038/ncomms15208 PMC5436223

[63] Reddy HK , Graña X , Dhanasekaran DN , Litvin J , Reddy EP . Requirement of Cdk4 for v‐Ha‐ras‐induced breast tumorigenesis and activation of the v‐ras‐induced senescence program by the R24C mutation. Genes Cancer. 2010;1(1):69‐80. 20634902 10.1177/1947601909358105 PMC2904236

[64] Kim YH , Park TJ . Cellular senescence in cancer. BMB reports. 2019;52(1):42‐46. 30526772 10.5483/BMBRep.2019.52.1.295 PMC6386235

[65] Acosta JC , O'Loghlen A , Banito A , et al. Chemokine signaling via the CXCR2 receptor reinforces senescence. Cell. 2008;133(6):1006‐1018. 18555777 10.1016/j.cell.2008.03.038

[66] Kuilman T , Michaloglou C , Vredeveld LC , et al. Oncogene‐induced senescence relayed by an interleukin‐dependent inflammatory network. Cell. 2008;133(6):1019‐1031. 18555778 10.1016/j.cell.2008.03.039

[67] Goel S , DeCristo MJ , Watt AC , et al. CDK4/6 inhibition triggers anti‐tumour immunity. Nature. 2017;548(7668):471‐475. 28813415 10.1038/nature23465 PMC5570667

[68] Ruscetti M , Morris JPt , Mezzadra R , et al. Senescence‐Induced Vascular Remodeling Creates Therapeutic Vulnerabilities in Pancreas Cancer. Cell. 2020;181(2):424‐441.e21. 32234521 10.1016/j.cell.2020.03.008 PMC7278897

[69] Kim JJ , Lee SB , Park JK , Yoo YD . TNF‐alpha‐induced ROS production triggering apoptosis is directly linked to Romo1 and Bcl‐X(L). Cell Death Differ. 2010;17(9):1420‐1434. 20203691 10.1038/cdd.2010.19

[70] Regis G , Icardi L , Conti L , et al. IL‐6, but not IFN‐gamma, triggers apoptosis and inhibits in vivo growth of human malignant T cells on STAT3 silencing. Leukemia. 2009;23(11):2102‐2108. 19626047 10.1038/leu.2009.139

[71] Tasdemir N , Banito A , Roe JS , et al. BRD4 connects enhancer remodeling to senescence immune surveillance. Cancer Discov. 2016;6(6):612‐629. 27099234 10.1158/2159-8290.CD-16-0217 PMC4893996

[72] Ventura A , Kirsch DG , McLaughlin ME , et al. Restoration of p53 function leads to tumour regression in vivo. Nature. 2007;445(7128):661‐665. 17251932 10.1038/nature05541

[73] Xue W , Zender L , Miething C , et al. Senescence and tumour clearance is triggered by p53 restoration in murine liver carcinomas. Nature. 2007;445(7128):656‐660. 17251933 10.1038/nature05529 PMC4601097

[74] Chiang DY , Villanueva A , Hoshida Y , et al. Focal gains of VEGFA and molecular classification of hepatocellular carcinoma. Cancer Res. 2008;68(16):6779‐6788. 18701503 10.1158/0008-5472.CAN-08-0742 PMC2587454

[75] Llovet JM , Kelley RK , Villanueva A , et al. Hepatocellular carcinoma. Nat Rev Dis Primers. 2021;7(1):6. 33479224 10.1038/s41572-020-00240-3 PMC13537433

[76] Reimann M , Lee S , Loddenkemper C , et al. Tumor stroma‐derived TGF‐beta limits myc‐driven lymphomagenesis via Suv39h1‐dependent senescence. Cancer Cell. 2010;17(3):262‐272. 20227040 10.1016/j.ccr.2009.12.043

[77] Lujambio A , Akkari L , Simon J , et al. Non‐cell‐autonomous tumor suppression by p53. Cell. 2013;153(2):449‐460. 23562644 10.1016/j.cell.2013.03.020 PMC3702034

[78] Sturmlechner I , Zhang C , Sine CC , et al. p21 produces a bioactive secretome that places stressed cells under immunosurveillance. Science. 2021;374(6567):eabb3420. 34709885 10.1126/science.abb3420 PMC8985214

[79] Ruscetti M , Leibold J , Bott MJ , et al. NK cell‐mediated cytotoxicity contributes to tumor control by a cytostatic drug combination. Science. 2018;362(6421):1416‐1422. 30573629 10.1126/science.aas9090 PMC6711172

[80] Heckler M , Ali LR , Clancy‐Thompson E , et al. Inhibition of CDK4/6 promotes CD8 T‐cell memory formation. Cancer Discov. 2021;11(10):2564‐2581. 33941591 10.1158/2159-8290.CD-20-1540 PMC8487897

[81] McHugh D , Gil J . Senescence and aging: causes, consequences, and therapeutic avenues. J Cell Biol. 2018;217(1):65‐77. 29114066 10.1083/jcb.201708092 PMC5748990

[82] Calcinotto A , Kohli J , Zagato E , Pellegrini L , Demaria M , Alimonti A . Cellular senescence: aging, cancer, and injury. Physiol Rev. 2019;99(2):1047‐1078. 30648461 10.1152/physrev.00020.2018

[83] Donnini S , Monti M , Castagnini C , et al. Pyrazolo‐pyrimidine‐derived c‐Src inhibitor reduces angiogenesis and survival of squamous carcinoma cells by suppressing vascular endothelial growth factor production and signaling. Int J Cancer. 2007;120(5):995‐1004. 17131343 10.1002/ijc.22410

[84] Khalilgharibi N , Mao Y . To form and function: on the role of basement membrane mechanics in tissue development, homeostasis and disease. Open biology. 2021;11(2):200360. 33593159 10.1098/rsob.200360 PMC8061686

[85] Guccini I , Revandkar A , D'Ambrosio M , et al. Senescence reprogramming by TIMP1 deficiency promotes prostate cancer metastasis. Cancer Cell. 2021;39(1):68‐82.e9. 33186519 10.1016/j.ccell.2020.10.012

[86] Waugh DJ , Wilson C . The interleukin‐8 pathway in cancer. Clinical cancer research : an official journal of the American Association for Cancer Research. 2008;14(21):6735‐6741. 18980965 10.1158/1078-0432.CCR-07-4843

[87] Wang L , Tang C , Cao H , et al. Activation of IL‐8 via PI3K/Akt‐dependent pathway is involved in leptin‐mediated epithelial‐mesenchymal transition in human breast cancer cells. Cancer Biol Ther. 2015;16(8):1220‐1230. 26121010 10.1080/15384047.2015.1056409 PMC4622725

[88] Watanabe S , Kawamoto S , Ohtani N , Hara E . Impact of senescence‐associated secretory phenotype and its potential as a therapeutic target for senescence‐associated diseases. Cancer Sci. 2017;108(4):563‐569. 28165648 10.1111/cas.13184 PMC5406532

[89] Pereira BI , Devine OP , Vukmanovic‐Stejic M , et al. Senescent cells evade immune clearance via HLA‐E‐mediated NK and CD8(+) T cell inhibition. Nat Commun. 2019;10(1):2387. 31160572 10.1038/s41467-019-10335-5 PMC6547655

[90] Milanovic M , Fan DNY , Belenki D , et al. Senescence‐associated reprogramming promotes cancer stemness. Nature. 2018;553(7686):96‐100. 29258294 10.1038/nature25167

[91] Lee S , Schmitt CA . The dynamic nature of senescence in cancer. Nat Cell Biol. 2019;21(1):94‐101. 30602768 10.1038/s41556-018-0249-2

[92] Patel PL , Suram A , Mirani N , Bischof O , Herbig U . Derepression of hTERT gene expression promotes escape from oncogene‐induced cellular senescence. Proc Nat Acad Sci USA. 2016;113(34):E5024‐E5033. 27503890 10.1073/pnas.1602379113 PMC5003242

[93] Benítez S , Cordero A , Santamaría PG , et al. RANK links senescence to stemness in the mammary epithelia, delaying tumor onset but increasing tumor aggressiveness. Dev Cell. 2021;56(12):1727‐1741.e7. 34004159 10.1016/j.devcel.2021.04.022 PMC8221814

[94] Galanos P , Vougas K , Walter D , et al. Chronic p53‐independent p21 expression causes genomic instability by deregulating replication licensing. Nat Cell Biol. 2016;18(7):777‐789. 27323328 10.1038/ncb3378 PMC6535144

[95] George AJ , Ritter MA . Thymic involution with ageing: obsolescence or good housekeeping? Immunol Today. 1996;17(6):267‐272. 8962629 10.1016/0167-5699(96)80543-3

[96] Franceschi C , Bonafè M , Valensin S , et al. Inflamm‐aging. An evolutionary perspective on immunosenescence. Ann NY Acad Sci. 2000;908:244‐254. 10911963 10.1111/j.1749-6632.2000.tb06651.x

[97] Fulop T , Larbi A , Dupuis G , et al. Immunosenescence and Inflamm‐Aging As Two Sides of the Same Coin: Friends or Foes? Front Immunol. 2017;8:1960. 29375577 10.3389/fimmu.2017.01960 PMC5767595

[98] Campisi J , Kapahi P , Lithgow GJ , Melov S , Newman JC , Verdin E . From discoveries in ageing research to therapeutics for healthy ageing. Nature. 2019;571(7764):183‐192. 31292558 10.1038/s41586-019-1365-2 PMC7205183

[99] Palmer S , Albergante L , Blackburn CC , Newman TJ . Thymic involution and rising disease incidence with age. Proc Nat Acad Sci USA. 2018;115(8):1883‐1888. 29432166 10.1073/pnas.1714478115 PMC5828591

[100] Liu X , Hoft DF , Peng G . Senescent T cells within suppressive tumor microenvironments: emerging target for tumor immunotherapy. J Clin Invest. 2020;130(3):1073‐1083. 32118585 10.1172/JCI133679 PMC7269563

[101] Vang T , Torgersen KM , Sundvold V , et al. Activation of the COOH‐terminal Src kinase (Csk) by cAMP‐dependent protein kinase inhibits signaling through the T cell receptor. J Exp Med. 2001;193(4):497‐507. 11181701 10.1084/jem.193.4.497 PMC2195911

[102] Sitkovsky MV , Kjaergaard J , Lukashev D , Ohta A . Hypoxia‐adenosinergic immunosuppression: tumor protection by T regulatory cells and cancerous tissue hypoxia. Clinical cancer research : an official journal of the American Association for Cancer Research. 2008;14(19):5947‐5952. 18829471 10.1158/1078-0432.CCR-08-0229

[103] Ye J , Huang X , Hsueh EC , et al. Human regulatory T cells induce T‐lymphocyte senescence. Blood. 2012;120(10):2021‐2031. 22723548 10.1182/blood-2012-03-416040 PMC3437594

[104] Liu X , Mo W , Ye J , et al. Regulatory T cells trigger effector T cell DNA damage and senescence caused by metabolic competition. Nat Commun. 2018;9(1):249. 29339767 10.1038/s41467-017-02689-5 PMC5770447

[105] Lanna A , Henson SM , Escors D , Akbar AN . The kinase p38 activated by the metabolic regulator AMPK and scaffold TAB1 drives the senescence of human T cells. Nat Immunol. 2014;15(10):965‐972. 25151490 10.1038/ni.2981 PMC4190666

[106] Das R , Ponnappan S , Ponnappan U . Redox regulation of the proteasome in T lymphocytes during aging. Free Radical Biol Med. 2007;42(4):541‐551. 17275686 10.1016/j.freeradbiomed.2006.11.020 PMC1858653

[107] Salminen A . Activation of immunosuppressive network in the aging process. Ageing Res Rev. 2020;57:100998. 31838128 10.1016/j.arr.2019.100998

[108] Maggiorani D , Le O , Lisi V , et al. Senescence drives immunotherapy resistance by inducing an immunosuppressive tumor microenvironment. Nat Commun. 2024;15(1):2435. 38499573 10.1038/s41467-024-46769-9 PMC10948808

[109] Nikolich‐Žugich J . The twilight of immunity: emerging concepts in aging of the immune system. Nat Immunol. 2018;19(1):10‐19. 29242543 10.1038/s41590-017-0006-x

[110] Chinn IK , Blackburn CC , Manley NR , Sempowski GD . Changes in primary lymphoid organs with aging. Semin Immunol. 2012;24(5):309‐320. 22559987 10.1016/j.smim.2012.04.005 PMC3415579

[111] Palmer DB . The effect of age on thymic function. Front Immunol. 2013;4:316. 24109481 10.3389/fimmu.2013.00316 PMC3791471

[112] Goronzy JJ , Weyand CM . Successful and maladaptive T cell aging. Immunity. 2017;46(3):364‐378. 28329703 10.1016/j.immuni.2017.03.010 PMC5433436

[113] Pawelec G . Immunosenescence and cancer. Biogerontology. 2017;18(4):717‐721. 28220304 10.1007/s10522-017-9682-z

[114] Fukushima Y , Minato N , Hattori M . The impact of senescence‐associated T cells on immunosenescence and age‐related disorders. Inflammation and regeneration. 2018;38:24. 30603051 10.1186/s41232-018-0082-9 PMC6304761

[115] Kurachi M , Barnitz RA , Yosef N , et al. The transcription factor BATF operates as an essential differentiation checkpoint in early effector CD8+ T cells. Nat Immunol. 2014;15(4):373‐383. 24584090 10.1038/ni.2834 PMC4000237

[116] Pereira BI , De Maeyer RPH , Covre LP , et al. Sestrins induce natural killer function in senescent‐like CD8(+) T cells. Nat Immunol. 2020;21(6):684‐694. 32231301 10.1038/s41590-020-0643-3 PMC10249464

[117] Björkström NK , Béziat V , Cichocki F , et al. CD8 T cells express randomly selected KIRs with distinct specificities compared with NK cells. Blood. 2012;120(17):3455‐3465. 22968455 10.1182/blood-2012-03-416867 PMC3482857

[118] Britanova OV , Putintseva EV , Shugay M , et al. Age‐related decrease in TCR repertoire diversity measured with deep and normalized sequence profiling. Journal of immunology (Baltimore, Md : 1950). 2014;192(6):2689‐2698. 24510963 10.4049/jimmunol.1302064

[119] Yang OO , Lin H , Dagarag M , Ng HL , Effros RB , Uittenbogaart CH . Decreased perforin and granzyme B expression in senescent HIV‐1‐specific cytotoxic T lymphocytes. Virology. 2005;332(1):16‐19. 15661136 10.1016/j.virol.2004.11.028

[120] Debacq‐Chainiaux F , Erusalimsky JD , Campisi J , Toussaint O . Protocols to detect senescence‐associated beta‐galactosidase (SA‐betagal) activity, a biomarker of senescent cells in culture and in vivo. Nat Protoc. 2009;4(12):1798‐1806. 20010931 10.1038/nprot.2009.191

[121] Martínez‐Zamudio RI , Dewald HK , Vasilopoulos T , Gittens‐Williams L , Fitzgerald‐Bocarsly P , Herbig U . Senescence‐associated β‐galactosidase reveals the abundance of senescent CD8+ T cells in aging humans. Aging Cell. 2021;20(5):e13344. 33939265 10.1111/acel.13344 PMC8135084

[122] Wang B , Han J , Elisseeff JH , Demaria M . The senescence‐associated secretory phenotype and its physiological and pathological implications. Nat Rev Mol Cell Biol. 2024;25(12):958‐978. 10.1038/s41580-024-00727-x 38654098

[123] Parish ST , Wu JE , Effros RB . Modulation of T lymphocyte replicative senescence via TNF‐{alpha} inhibition: role of caspase‐3. Journal of immunology (Baltimore, Md : 1950). 2009;182(7):4237‐4243. 19299722 10.4049/jimmunol.0803449 PMC3773494

[124] Herranz N , Gil J . Mechanisms and functions of cellular senescence. J Clin Invest. 2018;128(4):1238‐1246. 29608137 10.1172/JCI95148 PMC5873888

[125] Henson SM , Lanna A , Riddell NE , et al. p38 signaling inhibits mTORC1‐independent autophagy in senescent human CD8⁺ T cells. J Clin Invest. 2014;124(9):4004‐4016. 25083993 10.1172/JCI75051 PMC4151208

[126] Henson SM , Macaulay R , Riddell NE , Nunn CJ , Akbar AN . Blockade of PD‐1 or p38 MAP kinase signaling enhances senescent human CD8(+) T‐cell proliferation by distinct pathways. Eur J Immunol. 2015;45(5):1441‐1451. 25707450 10.1002/eji.201445312

[127] Callender LA , Carroll EC , Beal RWJ , et al. Human CD8(+) EMRA T cells display a senescence‐associated secretory phenotype regulated by p38 MAPK. Aging Cell. 2018;17(1):e12675. 29024417 10.1111/acel.12675 PMC5770853

[128] Aldinucci D , Borghese C , Casagrande N . The CCL5/CCR5 axis in cancer progression. Cancers. 2020;12(7):1765. 32630699 10.3390/cancers12071765 PMC7407580

[129] Ruhland MK , Loza AJ , Capietto AH , et al. Stromal senescence establishes an immunosuppressive microenvironment that drives tumorigenesis. Nat Commun. 2016;7:11762. 27272654 10.1038/ncomms11762 PMC4899869

[130] Nakamura K , Kassem S , Cleynen A , et al. Dysregulated IL‐18 is a key driver of immunosuppression and a possible therapeutic target in the multiple myeloma microenvironment. Cancer Cell. 2018;33(4):634‐648.e5. 29551594 10.1016/j.ccell.2018.02.007

[131] Castro F , Cardoso AP , Gonçalves RM , Serre K , Oliveira MJ . Interferon‐gamma at the crossroads of tumor immune surveillance or evasion. Front Immunol. 2018;9:847. 29780381 10.3389/fimmu.2018.00847 PMC5945880

[132] Sharma S , Dominguez AL , Lustgarten J . High accumulation of T regulatory cells prevents the activation of immune responses in aged animals. Journal of immunology (Baltimore, Md : 1950). 2006;177(12):8348‐8355. 17142731 10.4049/jimmunol.177.12.8348

[133] Thomas DC , Mellanby RJ , Phillips JM , Cooke A . An early age‐related increase in the frequency of CD4+ Foxp3+ cells in BDC2.5NOD mice. Immunology. 2007;121(4):565‐576. 17437531 10.1111/j.1365-2567.2007.02604.x PMC2265971

[134] Mittelbrunn M , Kroemer G . Hallmarks of T cell aging. Nat Immunol. 2021;22(6):687‐698. 33986548 10.1038/s41590-021-00927-z

[135] Stranks AJ , Hansen AL , Panse I , et al. Autophagy controls acquisition of aging features in macrophages. Journal of innate immunity. 2015;7(4):375‐391. 25764971 10.1159/000370112 PMC4386145

[136] Wong CK , Smith CA , Sakamoto K , Kaminski N , Koff JL , Goldstein DR . Aging impairs alveolar macrophage phagocytosis and increases influenza‐induced mortality in mice. Journal of immunology (Baltimore, Md : 1950). 2017;199(3):1060‐1068. 28646038 10.4049/jimmunol.1700397 PMC5557035

[137] Lumeng CN , Liu J , Geletka L , et al. Aging is associated with an increase in T cells and inflammatory macrophages in visceral adipose tissue. Journal of immunology (Baltimore, Md : 1950). 2011;187(12):6208‐6216. 22075699 10.4049/jimmunol.1102188 PMC3237772

[138] Fontana L , Zhao E , Amir M , Dong H , Tanaka K , Czaja MJ . Aging promotes the development of diet‐induced murine steatohepatitis but not steatosis. Hepatology (Baltimore, Md). 2013;57(3):995‐1004. 10.1002/hep.26099 PMC3566282 23081825

[139] Jackaman C , Radley‐Crabb HG , Soffe Z , Shavlakadze T , Grounds MD , Nelson DJ . Targeting macrophages rescues age‐related immune deficiencies in C57BL/6J geriatric mice. Aging Cell. 2013;12(3):345‐357. 23442123 10.1111/acel.12062

[140] Gon Y , Hashimoto S , Hayashi S , Koura T , Matsumoto K , Horie T . Lower serum concentrations of cytokines in elderly patients with pneumonia and the impaired production of cytokines by peripheral blood monocytes in the elderly. Clin Exp Immunol. 1996;106(1):120‐126. 8870709

[141] Plowden J , Renshaw‐Hoelscher M , Gangappa S , Engleman C , Katz JM , Sambhara S . Impaired antigen‐induced CD8+ T cell clonal expansion in aging is due to defects in antigen presenting cell function. Cell Immunol. 2004;229(2):86‐92. 15474523 10.1016/j.cellimm.2004.07.001

[142] van Duin D , Allore HG , Mohanty S , et al. Prevaccine determination of the expression of costimulatory B7 molecules in activated monocytes predicts influenza vaccine responses in young and older adults. J Infect Dis. 2007;195(11):1590‐1597. 17471428 10.1086/516788

[143] Minhas PS , Latif‐Hernandez A , McReynolds MR , et al. Restoring metabolism of myeloid cells reverses cognitive decline in ageing. Nature. 2021;590(7844):122‐128. 33473210 10.1038/s41586-020-03160-0 PMC8274816

[144] Gomez CR , Nomellini V , Faunce DE , Kovacs EJ . Innate immunity and aging. Exp Gerontol. 2008;43(8):718‐728. 18586079 10.1016/j.exger.2008.05.0168.05.016 PMC2564282

[145] Li X , Li C , Zhang W , Wang Y , Qian P , Huang H . Inflammation and aging: signaling pathways and intervention therapies. Signal transduction and targeted therapy. 2023;8(1):239. 37291105 10.1038/s41392-023-01502-8 PMC10248351

[146] Niwa Y , Kasama T , Miyachi Y , Kanoh T . Neutrophil chemotaxis, phagocytosis and parameters of reactive oxygen species in human aging: cross‐sectional and longitudinal studies. Life Sci. 1989;44(22):1655‐1664. 2733545 10.1016/0024-3205(89)90482-7

[147] Liles WC , Kiener PA , Ledbetter JA , Aruffo A , Klebanoff SJ . Differential expression of Fas (CD95) and Fas ligand on normal human phagocytes: implications for the regulation of apoptosis in neutrophils. J Exp Med. 1996;184(2):429‐440. 8760796 10.1084/jem.184.2.429 PMC2192712

[148] Butcher S , Chahel H , Lord JM . Review article: ageing and the neutrophil: no appetite for killing? Immunology. 2000;100(4):411‐416. 10929066 10.1046/j.1365-2567.2000.00079.x PMC2327031

[149] Hazeldine J , Harris P , Chapple IL , et al. Impaired neutrophil extracellular trap formation: a novel defect in the innate immune system of aged individuals. Aging Cell. 2014;13(4):690‐698. 24779584 10.1111/acel.12222 PMC4326942

[150] Qian F , Guo X , Wang X , et al. Reduced bioenergetics and toll‐like receptor 1 function in human polymorphonuclear leukocytes in aging. Aging. 2014;6(2):131‐139. 24595889 10.18632/aging.100642 PMC3969281

[151] Dubey M , Nagarkoti S , Awasthi D , et al. Nitric oxide‐mediated apoptosis of neutrophils through caspase‐8 and caspase‐3‐dependent mechanism. Cell Death Dis. 2016;7(9):e2348. 27584786 10.1038/cddis.2016.248 PMC5059853

[152] Grecian R , Whyte MKB , Walmsley SR . The role of neutrophils in cancer. Br Med Bull. 2018;128(1):5‐14. 30137312 10.1093/bmb/ldy029 PMC6289220

[153] Xue R , Zhang Q , Cao Q , et al. Liver tumour immune microenvironment subtypes and neutrophil heterogeneity. Nature. 2022;612(7938):141‐147. 36352227 10.1038/s41586-022-05400-x

[154] Fridlender ZG , Albelda SM . Tumor‐associated neutrophils: friend or foe? Carcinogenesis. 2012;33(5):949‐955. 22425643 10.1093/carcin/bgs123

[155] Wang Y , Ding Y , Guo N , Wang S. MDSCs : Key criminals of tumor pre‐metastatic niche formation. Front Immunol. 2019;10:172. 30792719 10.3389/fimmu.2019.00172 PMC6374299

[156] Lian J , Yue Y , Yu W , Zhang Y . Immunosenescence: a key player in cancer development. J Hematol Oncol. 2020;13(1):151. 33168037 10.1186/s13045-020-00986-z PMC7653700

[157] Enioutina EY , Bareyan D , Daynes RA . A role for immature myeloid cells in immune senescence. Journal of immunology (Baltimore, Md : 1950). 2011;186(2):697‐707. 21148798 10.4049/jimmunol.1002987

[158] Hurez V , Daniel BJ , Sun L , et al. Mitigating age‐related immune dysfunction heightens the efficacy of tumor immunotherapy in aged mice. Cancer Res. 2012;72(8):2089‐2099. 22496463 10.1158/0008-5472.CAN-11-3019 PMC3328641

[159] Okuma A , Hanyu A , Watanabe S , Hara E . p16(Ink4a) and p21(Cip1/Waf1) promote tumour growth by enhancing myeloid‐derived suppressor cells chemotaxis. Nat Commun. 2017;8(1):2050. 29234059 10.1038/s41467-017-02281-x PMC5727112

[160] Wu SY , Fu T , Jiang YZ , Shao ZM . Natural killer cells in cancer biology and therapy. Mol Cancer. 2020;19(1):120. 32762681 10.1186/s12943-020-01238-x PMC7409673

[161] Sansoni P , Cossarizza A , Brianti V , et al. Lymphocyte subsets and natural killer cell activity in healthy old people and centenarians. Blood. 1993;82(9):2767‐2773. 8219229

[162] Campos C , López N , Pera A , et al. Expression of NKp30, NKp46 and DNAM‐1 activating receptors on resting and IL‐2 activated NK cells from healthy donors according to CMV‐serostatus and age. Biogerontology. 2015;16(5):671‐683. 25991472 10.1007/s10522-015-9581-0

[163] Martín‐Fontecha A , Thomsen LL , Brett S , et al. Induced recruitment of NK cells to lymph nodes provides IFN‐gamma for T(H)1 priming. Nat Immunol. 2004;5(12):1260‐1265. 15531883 10.1038/ni1138

[164] Wolf NK , Kissiov DU , Raulet DH . Roles of natural killer cells in immunity to cancer, and applications to immunotherapy. Nat Rev Immunol. 2023;23(2):90‐105. 35637393 10.1038/s41577-022-00732-1

[165] Di Micco R , Krizhanovsky V , Baker D , d'Adda di Fagagna F . Cellular senescence in ageing: from mechanisms to therapeutic opportunities. Nat Rev Mol Cell Biol. 2021;22(2):75‐95. 33328614 10.1038/s41580-020-00314-w PMC8344376

[166] Chen HA , Ho YJ , Mezzadra R , et al. Senescence rewires microenvironment sensing to facilitate antitumor immunity. Cancer Discov. 2023;13(2):432‐453. 36302222 10.1158/2159-8290.CD-22-0528 PMC9901536

[167] Marin I , Boix O , Garcia‐Garijo A , et al. Cellular senescence is immunogenic and promotes antitumor immunity. Cancer Discov. 2023;13(2):410‐431. 36302218 10.1158/2159-8290.CD-22-0523 PMC7614152

[168] Prieto LI , Sturmlechner I , Goronzy JJ , Baker DJ . Senescent cells as thermostats of antitumor immunity. Sci Transl Med. 2023;15(699):eadg7291. 37285401 10.1126/scitranslmed.adg7291 PMC10362799

[169] Shahbandi A , Chiu FY , Ungerleider NA , et al. Breast cancer cells survive chemotherapy by activating targetable immune‐modulatory programs characterized by PD‐L1 or CD80. Nature cancer. 2022;3(12):1513‐1533. 36482233 10.1038/s43018-022-00466-y PMC9923777

[170] Wang TW , Johmura Y , Suzuki N , et al. Blocking PD‐L1‐PD‐1 improves senescence surveillance and ageing phenotypes. Nature. 2022;611(7935):358‐364. 36323784 10.1038/s41586-022-05388-4

[171] Wang L , Lankhorst L , Bernards R . Exploiting senescence for the treatment of cancer. Nat Rev Cancer. 2022;22(6):340‐355. 35241831 10.1038/s41568-022-00450-9

[172] Chang J , Wang Y , Shao L , et al. Clearance of senescent cells by ABT263 rejuvenates aged hematopoietic stem cells in mice. Nat Med. 2016;22(1):78‐83. 26657143 10.1038/nm.4010 PMC4762215

[173] Adams JM , Cory S . The BCL‐2 arbiters of apoptosis and their growing role as cancer targets. Cell Death Differ. 2018;25(1):27‐36. 29099483 10.1038/cdd.2017.161 PMC5729526

[174] Mylonas KJ , O'Sullivan ED , Humphries D , et al. Cellular senescence inhibits renal regeneration after injury in mice, with senolytic treatment promoting repair. Sci Transl Med. 2021;13(594):eabb0203. 34011625 10.1126/scitranslmed.abb0203

[175] Fung AS , Wu L , Tannock IF . Concurrent and sequential administration of chemotherapy and the mammalian target of rapamycin inhibitor temsirolimus in human cancer cells and xenografts. Clinical cancer research : an official journal of the American Association for Cancer Research. 2009;15(17):5389‐5395. 19706800 10.1158/1078-0432.CCR-08-3007

[176] Kucheryavenko O , Nelson G , von Zglinicki T , Korolchuk VI , Carroll B . The mTORC1‐autophagy pathway is a target for senescent cell elimination. Biogerontology. 2019;20(3):331‐335. 30798505 10.1007/s10522-019-09802-9 PMC6535413

[177] Wang C , Vegna S , Jin H , et al. Inducing and exploiting vulnerabilities for the treatment of liver cancer. Nature. 2019;574(7777):268‐272. 31578521 10.1038/s41586-019-1607-3 PMC6858884

[178] Baar MP , Brandt RMC , Putavet DA , et al. Targeted apoptosis of senescent cells restores tissue homeostasis in response to chemotoxicity and aging. Cell. 2017;169(1):132‐147.e16. 28340339 10.1016/j.cell.2017.02.031 PMC5556182

[179] Jeon OH , Kim C , Laberge RM , et al. Local clearance of senescent cells attenuates the development of post‐traumatic osteoarthritis and creates a pro‐regenerative environment. Nat Med. 2017;23(6):775‐781. 28436958 10.1038/nm.4324 PMC5785239

[180] Lu J , Qian Y , Altieri M , et al. Hijacking the E3 ubiquitin ligase cereblon to efficiently target BRD4. Chem Biol. 2015;22(6):755‐763. 26051217 10.1016/j.chembiol.2015.05.009 PMC4475452

[181] Wakita M , Takahashi A , Sano O , et al. A BET family protein degrader provokes senolysis by targeting NHEJ and autophagy in senescent cells. Nat Commun. 2020;11(1):1935. 32321921 10.1038/s41467-020-15719-6 PMC7176673

[182] Guerrero A , Herranz N , Sun B , et al. Cardiac glycosides are broad‐spectrum senolytics. Nature metabolism. 2019;1(11):1074‐1088. 10.1038/s42255-019-0122-z PMC6887543 31799499

[183] Triana‐Martínez F , Picallos‐Rabina P , Da Silva‐Álvarez S , et al. Identification and characterization of Cardiac Glycosides as senolytic compounds. Nat Commun. 2019;10(1):4731. 31636264 10.1038/s41467-019-12888-x PMC6803708

[184] Amor C , Feucht J , Leibold J , et al. Senolytic CAR T cells reverse senescence‐associated pathologies. Nature. 2020;583(7814):127‐132. 32555459 10.1038/s41586-020-2403-9 PMC7583560

[185] Arora S , Thompson PJ , Wang Y , et al. Invariant natural killer T cells coordinate removal of senescent cells. Med (New York, NY). 2021;2(8):938‐950. 10.1016/j.medj.2021.04.014 PMC8491998 34617070

[186] Mendelsohn AR , Larrick JW . Antiaging vaccines targeting senescent cells. Rejuvenation Res. 2022;25(1):39‐45. 35081729 10.1089/rej.2022.0008

[187] Moiseeva O , Deschênes‐Simard X , St‐Germain E , et al. Metformin inhibits the senescence‐associated secretory phenotype by interfering with IKK/NF‐κB activation. Aging Cell. 2013;12(3):489‐498. 23521863 10.1111/acel.12075

[188] Herranz N , Gallage S , Mellone M , et al. mTOR regulates MAPKAPK2 translation to control the senescence‐associated secretory phenotype. Nat Cell Biol. 2015;17(9):1205‐1217. 26280535 10.1038/ncb3225 PMC4589897

[189] Xu M , Tchkonia T , Ding H , et al. JAK inhibition alleviates the cellular senescence‐associated secretory phenotype and frailty in old age. Proc Nat Acad Sci USA. 2015;112(46):E6301‐E6310. 26578790 10.1073/pnas.1515386112 PMC4655580

[190] Chen R , Chen B . Siltuximab (CNTO 328): a promising option for human malignancies. Drug Des Dev Ther. 2015;9:3455‐3458. 10.2147/DDDT.S86438 PMC4494175 26170629

[191] Schenk KM , Reuss JE , Choquette K , Spira AI . A review of canakinumab and its therapeutic potential for non‐small cell lung cancer. Anticancer Drugs. 2019;30(9):879‐885. 31503012 10.1097/CAD.0000000000000832

[192] Toillon RA , Magné N , Laïos I , et al. Estrogens decrease gamma‐ray‐induced senescence and maintain cell cycle progression in breast cancer cells independently of p53. International journal of radiation oncology *, biology, physics*. 2007;67(4):1187‐1200. 10.1016/j.ijrobp.2006.11.040 17336220

[193] Poulsen RC , Watts AC , Murphy RJ , Snelling SJ , Carr AJ , Hulley PA . Glucocorticoids induce senescence in primary human tenocytes by inhibition of sirtuin 1 and activation of the p53/p21 pathway: in vivo and in vitro evidence. Ann Rheum Dis. 2014;73(7):1405‐1413. 23727633 10.1136/annrheumdis-2012-203146 PMC4078757

[194] Rais M , Wilson RM , Urbanski HF , Messaoudi I . Androgen supplementation improves some but not all aspects of immune senescence in aged male macaques. GeroScience. 2017;39(4):373‐384. 28616771 10.1007/s11357-017-9979-5 PMC5636781

[195] Yu S , Wang X , Geng P , et al. Melatonin regulates PARP1 to control the senescence‐associated secretory phenotype (SASP) in human fetal lung fibroblast cells. J Pineal Res. 2017;63(1). 10.1111/jpi.12405 28247536

[196] Ye J , Ma C , Hsueh EC , et al. Tumor‐derived γδ regulatory T cells suppress innate and adaptive immunity through the induction of immunosenescence. Journal of immunology (Baltimore, Md : 1950). 2013;190(5):2403‐2414. 23355732 10.4049/jimmunol.1202369 PMC3578061

[197] Ye J , Ma C , Hsueh EC , et al. TLR8 signaling enhances tumor immunity by preventing tumor‐induced T‐cell senescence. EMBO Mol Med. 2014;6(10):1294‐1311. 25231413 10.15252/emmm.201403918 PMC4287933

[198] Alizadeh D , Wong RA , Yang X , et al. IL15 enhances CAR‐T cell antitumor activity by reducing mTORC1 activity and preserving their stem cell memory phenotype. Cancer Immunol Res. 2019;7(5):759‐772. 30890531 10.1158/2326-6066.CIR-18-0466 PMC6687561

[199] Bharath LP , Agrawal M , McCambridge G , et al. Metformin enhances autophagy and normalizes mitochondrial function to alleviate aging‐associated inflammation. Cell Metab. 2020;32(1):44‐55.e6. 32402267 10.1016/j.cmet.2020.04.015 PMC7217133

[200] Hachmo Y , Hadanny A , Abu Hamed R , et al. Hyperbaric oxygen therapy increases telomere length and decreases immunosenescence in isolated blood cells: a prospective trial. Aging. 2020;12(22):22445‐22456. 33206062 10.18632/aging.202188 PMC7746357

[201] Boni A , Cogdill AP , Dang P , et al. Selective BRAFV600E inhibition enhances T‐cell recognition of melanoma without affecting lymphocyte function. Cancer Res. 2010;70(13):5213‐5219. 20551059 10.1158/0008-5472.CAN-10-0118

[202] Frederick DT , Piris A , Cogdill AP , et al. BRAF inhibition is associated with enhanced melanoma antigen expression and a more favorable tumor microenvironment in patients with metastatic melanoma. Clinical cancer research : an official journal of the American Association for Cancer Research. 2013;19(5):1225‐1231. 23307859 10.1158/1078-0432.CCR-12-1630 PMC3752683

[203] Manic G , Obrist F , Sistigu A , Vitale I . Trial watch: targeting ATM‐CHK2 and ATR‐CHK1 pathways for anticancer therapy. Molecular & cellular oncology. 2015;2(4):e1012976. 27308506 10.1080/23723556.2015.1012976 PMC4905354

[204] Demaria M , O'Leary MN , Chang J , et al. Cellular senescence promotes adverse effects of chemotherapy and cancer relapse. Cancer Discov. 2017;7(2):165‐176. 27979832 10.1158/2159-8290.CD-16-0241 PMC5296251

[205] Liu Y , Lomeli I , Kron SJ . Therapy‐induced cellular senescence: potentiating tumor elimination or driving cancer resistance and recurrence? Cells. 2024;13(15) 10.3390/cells13151281 PMC11312217 39120312

[206] Kirkland JL , Tchkonia T , Zhu Y , Niedernhofer LJ , Robbins PD . The clinical potential of senolytic drugs. J Am Geriatr Soc. 2017;65(10):2297‐2301. 28869295 10.1111/jgs.14969 PMC5641223

[207] Yosef R , Pilpel N , Tokarsky‐Amiel R , et al. Directed elimination of senescent cells by inhibition of BCL‐W and BCL‐XL. Nat Commun. 2016;7:11190. 27048913 10.1038/ncomms11190 PMC4823827

[208] Zhu Y , Tchkonia T , Fuhrmann‐Stroissnigg H , et al. Identification of a novel senolytic agent, navitoclax, targeting the Bcl‐2 family of anti‐apoptotic factors. Aging Cell. 2016;15(3):428‐435. 26711051 10.1111/acel.12445 PMC4854923

[209] Fleury H , Malaquin N , Tu V , et al. Exploiting interconnected synthetic lethal interactions between PARP inhibition and cancer cell reversible senescence. Nat Commun. 2019;10(1):2556. 31186408 10.1038/s41467-019-10460-1 PMC6560032

[210] Saleh T , Carpenter VJ , Tyutyunyk‐Massey L , et al. Clearance of therapy‐induced senescent tumor cells by the senolytic ABT‐263 via interference with BCL‐X(L) ‐BAX interaction. Molecular oncology. 2020;14(10):2504‐2519. 32652830 10.1002/1878-0261.12761 PMC7530780

[211] Kipps TJ , Eradat H , Grosicki S , et al. A phase 2 study of the BH3 mimetic BCL2 inhibitor navitoclax (ABT‐263) with or without rituximab, in previously untreated B‐cell chronic lymphocytic leukemia. Leuk Lymphoma. 2015;56(10):2826‐2833. 25797560 10.3109/10428194.2015.1030638 PMC4643417

[212] Kaefer A , Yang J , Noertersheuser P , et al. Mechanism‐based pharmacokinetic/pharmacodynamic meta‐analysis of navitoclax (ABT‐263) induced thrombocytopenia. Cancer Chemother Pharmacol. 2014;74(3):593‐602. 25053389 10.1007/s00280-014-2530-9

[213] González‐Gualda E , Pàez‐Ribes M , Lozano‐Torres B , et al. Galacto‐conjugation of Navitoclax as an efficient strategy to increase senolytic specificity and reduce platelet toxicity. Aging Cell. 2020;19(4):e13142. 32233024 10.1111/acel.13142 PMC7189993

[214] Galiana I , Lozano‐Torres B , Sancho M , et al. Preclinical antitumor efficacy of senescence‐inducing chemotherapy combined with a nanoSenolytic. Journal of controlled release : official journal of the Controlled Release Society. 2020;323:624‐634. 32376460 10.1016/j.jconrel.2020.04.045

[215] Estepa‐Fernández A , Alfonso M , Morellá‐Aucejo Á , et al. Senolysis reduces senescence in veins and cancer cell migration. Advanced Therapeutics. 2021;4(10):2100149.

[216] He Y , Zhang X , Chang J , et al. Using proteolysis‐targeting chimera technology to reduce navitoclax platelet toxicity and improve its senolytic activity. Nat Commun. 2020;11(1):1996. 32332723 10.1038/s41467-020-15838-0 PMC7181703

[217] Martin JC , Sims JR , Gupta A , et al. CDC7 kinase (DDK) inhibition disrupts DNA replication leading to mitotic catastrophe in Ewing sarcoma. Cell death discovery. 2022;8(1):85. 35220396 10.1038/s41420-022-00877-x PMC8882187

[218] Efeyan A , Serrano M . p53: guardian of the genome and policeman of the oncogenes. Cell cycle (Georgetown, Tex). 2007;6(9):1006‐1010. 17457049 10.4161/cc.6.9.4211

[219] Levine AJ , Oren M . The first 30 years of p53: growing ever more complex. Nat Rev Cancer. 2009;9(10):749‐758. 19776744 10.1038/nrc2723 PMC2771725

[220] Morgan RA , Dudley ME , Wunderlich JR , et al. Cancer regression in patients after transfer of genetically engineered lymphocytes. Science. 2006;314(5796):126‐129. 16946036 10.1126/science.1129003 PMC2267026

[221] Gorgoulis VG , Pratsinis H , Zacharatos P , et al. p53‐dependent ICAM‐1 overexpression in senescent human cells identified in atherosclerotic lesions. Lab Invest. 2005;85(4):502‐511. 15711569 10.1038/labinvest.3700241

[222] Cui H , Kong Y , Xu M , Zhang H . Notch3 functions as a tumor suppressor by controlling cellular senescence. Cancer Res. 2013;73(11):3451‐3459. 23610446 10.1158/0008-5472.CAN-12-3902 PMC3674178

[223] Althubiti M , Lezina L , Carrera S , et al. Characterization of novel markers of senescence and their prognostic potential in cancer. Cell Death Dis. 2014;5(11):e1528. 10.1038/cddis.2014.489 PMC4260747 25412306

[224] Hoare M , Ito Y , Kang TW , et al. NOTCH1 mediates a switch between two distinct secretomes during senescence. Nat Cell Biol. 2016;18(9):979‐992. 27525720 10.1038/ncb3397 PMC5008465

[225] Sagiv A , Burton DG , Moshayev Z , et al. NKG2D ligands mediate immunosurveillance of senescent cells. Aging. 2016;8(2):328‐344. 26878797 10.18632/aging.100897 PMC4789586

[226] Kim KM , Noh JH , Bodogai M , et al. Identification of senescent cell surface targetable protein DPP4. Genes Dev. 2017;31(15):1529‐1534. 28877934 10.1101/gad.302570.117 PMC5630018

[227] Harrison CN , Mesa RA , Kiladjian JJ , et al. Health‐related quality of life and symptoms in patients with myelofibrosis treated with ruxolitinib versus best available therapy. Br J Haematol. 2013;162(2):229‐239. 23672349 10.1111/bjh.12375

[228] Callender LA , Carroll EC , Bober EA , Henson SM . Divergent mechanisms of metabolic dysfunction drive fibroblast and T‐cell senescence. Ageing Res Rev. 2018;47:24‐30. 29902528 10.1016/j.arr.2018.06.001

[229] Zhang Y , Ertl HC . Aging: T cell metabolism within tumors. Aging. 2016;8(6):1163‐1164. 27282177 10.18632/aging.100979 PMC4931823

[230] Liu X , Hartman CL , Li L , et al. Reprogramming lipid metabolism prevents effector T cell senescence and enhances tumor immunotherapy. Sci Transl Med. 2021;13(587):eaaz6314. 33790024 10.1126/scitranslmed.aaz6314 PMC12040281

[231] Liu Z , Liang Q , Ren Y , et al. Immunosenescence: molecular mechanisms and diseases. Signal transduction and targeted therapy. 2023;8(1):200. 37179335 10.1038/s41392-023-01451-2 PMC10182360

[232] Ye J , Peng G . Controlling T cell senescence in the tumor microenvironment for tumor immunotherapy. Oncoimmunology. 2015;4(3):e994398. 25949919 10.4161/2162402X.2014.994398 PMC4404789

[233] Veyrat M , Durand S , Classe M , et al. Stimulation of the toll‐like receptor 3 promotes metabolic reprogramming in head and neck carcinoma cells. Oncotarget. 2016;7(50):82580‐82593. 27791989 10.18632/oncotarget.12892 PMC5347715

[234] Huang L , Xu H , Peng G . TLR‐mediated metabolic reprogramming in the tumor microenvironment: potential novel strategies for cancer immunotherapy. Cellular & molecular immunology. 2018;15(5):428‐437. 29553135 10.1038/cmi.2018.4 PMC6068099

[235] Davis T , Bagley MC , Dix MC , et al. Synthesis and in vivo activity of MK2 and MK2 substrate‐selective p38alpha(MAPK) inhibitors in Werner syndrome cells. Bioorg Med Chem Lett. 2007;17(24):6832‐6835. 17964780 10.1016/j.bmcl.2007.10.036

[236] Wright WE , Pereira‐Smith OM , Shay JW . Reversible cellular senescence: implications for immortalization of normal human diploid fibroblasts. Mol Cell Biol. 1989;9(7):3088‐3092. 2779554 10.1128/mcb.9.7.3088-3092.1989 PMC362778

[237] Akbar AN , Henson SM . Are senescence and exhaustion intertwined or unrelated processes that compromise immunity? Nat Rev Immunol. 2011;11(4):289‐295. 21436838 10.1038/nri2959

[238] Nardella C , Clohessy JG , Alimonti A , Pandolfi PP . Pro‐senescence therapy for cancer treatment. Nat Rev Cancer. 2011;11(7):503‐511. 21701512 10.1038/nrc3057

[239] Reiser J , Banerjee A . Effector, memory, and dysfunctional CD8(+) T cell fates in the antitumor immune response. J Immunol Res. 2016;2016:8941260. 27314056 10.1155/2016/8941260 PMC4893440

[240] Ono K , Han J . The p38 signal transduction pathway: activation and function. Cell Signal. 2000;12(1):1‐13. 10676842 10.1016/s0898-6568(99)00071-6

[241] Bang YJ , Im SA , Lee KW , et al. Randomized, double‐blind phase II trial with prospective classification by ATM protein level to evaluate the efficacy and tolerability of olaparib plus paclitaxel in patients with recurrent or metastatic gastric cancer. Journal of clinical oncology : official journal of the American Society of Clinical Oncology. 2015;33(33):3858‐3865. 26282658 10.1200/JCO.2014.60.0320

[242] Lanna A , Gomes DC , Muller‐Durovic B , et al. A sestrin‐dependent Erk‐Jnk‐p38 MAPK activation complex inhibits immunity during aging. Nat Immunol. 2017;18(3):354‐363. 28114291 10.1038/ni.3665 PMC5321575

[243] Wiley CD , Sharma R , Davis SS , et al. Oxylipin biosynthesis reinforces cellular senescence and allows detection of senolysis. Cell Metab. 2021;33(6):1124‐1136.e5. 33811820 10.1016/j.cmet.2021.03.008 PMC8501892

